# Urease inhibitors technologies as strategy to mitigate agricultural ammonia emissions and enhance the use efficiency of urea-based fertilizers

**DOI:** 10.1038/s41598-023-50061-z

**Published:** 2023-12-20

**Authors:** Adrianne Braga da Fonseca, César Santos, Ana Paula Pereira Nunes, Damiany Pádua Oliveira, Maria Elisa Araújo de Melo, Thalita Takayama, Bethânia Leite Mansur, Thales de Jesus Fernandes, Gilson do Carmo Alexandrino, Marcos Altomani Neves Dias, Douglas Guelfi

**Affiliations:** 1 Laboratory of Fertilizers Technologies-INNOVA FERT, Department of Soil Science, Federal University of Lavras-UFLA, P.O. Box 3037, Lavras, MG 37203-202 Brazil; 2 Departament of Statistic, Federal University of Lavras, Lavras, MG Brazil; 3 NUTRIEN, São Paulo, Brazil

**Keywords:** Plant sciences, Element cycles, Environmental impact

## Abstract

Experiments were conducted to evaluate the stability and degradation of NBPT under storage conditions and to quantify urease activity, ammonia losses by volatilization, and agronomic efficiency of urea treated with different urease inhibitors, measured in the field. Experiments included urea treated with 530 mg NBPT kg^−1^ (UNBPT) in contact with six P-sources (monoammonium phosphate-MAP; single superphosphate; triple superphosphate; P-Agrocote; P-Phusion; P-Policote), with two P-concentrations (30; 70%); the monitoring four N-technologies (SoILC; Limus; Nitrain; Anvol); and the application of conventional urea (U_GRAN_) or urea treated with urease inhibitors as topdressing in three maize fields, at three N rates. It is concluded that: the mixture of UNBPT and P-fertilizers is incompatible. When MAP granules were coated to control P-release (P-Agrocote), the degradation of NBPT was moderate (approximately 400 mg kg^−1^ at the end of the storage test). SoILC and Limus solvent technologies extended the NBPT half-life by up to 3.7 and 4.7 months, respectively. Under field, each inhibition technology reduced urease activity, and lowered the intensity of ammonia emission compared to U_GRAN_ by 50–62%. Our results show that the concentration of NBPT is reduced by up to 53.7% for mixing with phosphates. In addition, even with coatings, the storage of mixtures of urea with NBPT and phosphates should be for a time that does not reduce the efficiency of the inhibitor after application, and this time under laboratory conditions was 168 h. The reduction of NBPT concentration in urea is reduced even in isolated storage, our results showed that the half-life time is variable according to the formulation used, being 4.7, 3.7, 2.8 and 2.7 days for Limus, SoILC, Nitrain and Anvol, respectively. The results of these NBPT formulations in the field showed that the average losses by volatilization in the three areas were: 15%, 16%, 17%, 19% and 39% of the N applied, for SoILC, Anvol, Nitrain, Limus and urea, respectively. The rate of nitrogen application affected all agronomic variables, with varied effects in Ingaí. Even without N, yields were higher than 9200 kg ha^−1^ of grains. The increase in nitrogen rates resulted in linear increases in production and N removal in Luminárias and Ingaí, but in Lavras, production decreased above 95.6 kg ha^−1^ of N. The highest production in Lavras (13,772 kg ha^−1^ of grains) occurred with 100 kg ha^−1^ of N. The application of Anvol reduced the removal of N in Ingaí.

## Introduction

The world consumption of NPK fertilizers in 2020/2021 corresponded to 198.2 Mt, regarded as the highest amount since 2011^1^. Among these fertilizers, the demand for N is nearly 110 Mt. As for P and K, the demand corresponds to 49.6 Mt and 38.5 Mt, respectively. Thus, compared to other nutrients, the use of N as fertilizers in agriculture is the highest, and urea is the primary source^1^.

Nutritionally, N stands out as the nutrient most required by the crops. It assists in growth and increases plants yields^2^. Owing to the tropical conditions, N undergoes several transformations in the system. The most important would be the ammonia emissions (NH_3_) to atmosphere^3^, particularly when urea is used without any associated technology^2–6^. The losses of NH_3_ by volatilization for conventional urea, in Brazil, range between 20 and 40% of the applied N. The N application rate for maize is usually 100 kg ha^−1^, and the urea ton costs nearly US$ 1200. Then, the NH_3_ losses can cost between US$ 54 and 107, or between US$ 54,000 and 107,000 in a 1000 ha^−1^ property.

Technologies that reduce N losses through, ammonia, and nitrous oxide emissions are strategies to be used in cleaner agriculture^2–7^. National policies that focus on good agricultural practices have been implemented as an attempt to minimize the adverse effects caused by volatilization and its environmental consequences. In 2001, the European Union (EU) adopted a directive to mitigate emissions of polluting gases, such as ammonia and nitrous oxide, regulating reductions of 30% of NH_3_ for some countries until 2030, relatively to 2005^8^.

Thus, researchers and fertilizer industries have studied and created alternatives to reduce greenhouse gas emissions and increase N efficiency. Such alternatives include treating urea with some additive and coating the granules of fertilizers^9,10^.

In the soil, the urease activity is essential for urea breakdown and N release in the soil, but increased enzymatic activity may cause high N losses in agriculture^11,12^. Thus, some products like urease inhibitors can inhibit the activity of the enzyme^6,13^. Currently, this group of technologies includes the association of different inhibitors in the same granule of fertilizer (such as Limus and Anvol technology)^13,14^ or with biocatalysts (Nitrain technology)^15^. However, there is a lack of information on the mechanisms involved in these technologies, and such knowledge is crucial for their proper functioning in the field.

Studies show that fertilizers treated with NBPT (*N*-(*n*-butyl) thiophosphoric triamide) can reduce up to 78% of NH_3_ emissions compared to urea^16^. However, the performance of the urease inhibitors depends on the type of solvent used in the additive^12^, storage conditions^17^ and factors related to the soil system, they are influenced by urease activity^18^, temperature^11^, pH^19^, and soil moistur^20^. The solvents can improve the molecule stability and protect it for more time in field^6,21–23^, but this depends on their properties, including pH, absence of water, and dissolution capacity.

Despite being an interesting strategy in the mitigation of N losses by volatilization, some particularities related to the formulation of urea treated with NBPT in the fertilizer industry still need to be understood and improved. For example, the mixture between NBPT and phosphate fertilizers which is often commercialized in the market. However, such mixture may present incompatibility in fertilizers based on a mixture of granules. Despite the relevance of this issue, the literature has almost no reports on the mechanisms involved in the possible inefficiency of NBPT in such conditions. Sha et al.^17^ evaluated the stability and efficiency of NBPT after storage with diammonium phosphate (DAP). The researchers demonstrated that the free acidity associated with this fertilizer was too harmful to NBPT, for promoting fast degradation of the molecule. Thus, conducting studies with other fertilizers is also needed. At the same time, strategies that allow using urea treated with NBPT and phosphate fertilizers should be evaluated. Such evaluation can be particularly relevant for phosphate fertilizers coated with polymers, which can prevent direct contact between NBPT and the granule of phosphate fertilizer.

Moreover, the storage conditions of urea treated with NBPT should be better explained. It is well known that high temperature, humidity, and product storage time can affect the use efficiency of treated urea in the field, thus affecting the ability of inhibitors to reduce NH_3_ losses^24^. Some improvements in urea treatment processes have been proposed, including the association of inhibitors^21,22^ and increasing the NBPT concentration in urea.

In summary, this research includes both laboratory studies and conducted under field conditions. Considering the many issues discussed about urea treated with NBPT, the hypotheses of the present study were: (1) the contact between NBPT and conventional fertilizers [Monoammonium Phosphate (MAP), Triple Superphosphate (TSP), and Single Superphosphate (SSP)] leads to NBPT degradation; (2) Phosphate fertilizers with associated technologies (Agrocote, Policote, and Phusion) can reduce or prevent NBPT degradation; (3) The varying inhibitor formulations can protect the NBPT molecule when urea is stored without mixing with phosphate; (4) The varying inhibitor technologies available on the market can reduce urease activity and mitigate NH_3_ losses in maize production areas; (5) The reduction of volatilization losses can influence the N accumulation in the plant at the flowering stage and increase maize yield.

To test our hypotheses, we firstly evaluated the effect of mixing urea treated with NBPT and conventional phosphate fertilizers and associated technologies in reducing the concentration of NBPT in urea. Then, the different technologies of urease inhibitors present on the market were stored, and the NBPT concentrations were evaluated at 30-day intervals. Lastly, the effects of different inhibitor technologies were evaluated under field conditions, and their ability to reduce urease activity and volatilization and influence N accumulation at flowering and maize yield.

## Results

### Storage compatibility of mixtures between urea treated with NBPT and phosphate fertilizers

**Figure 1:**
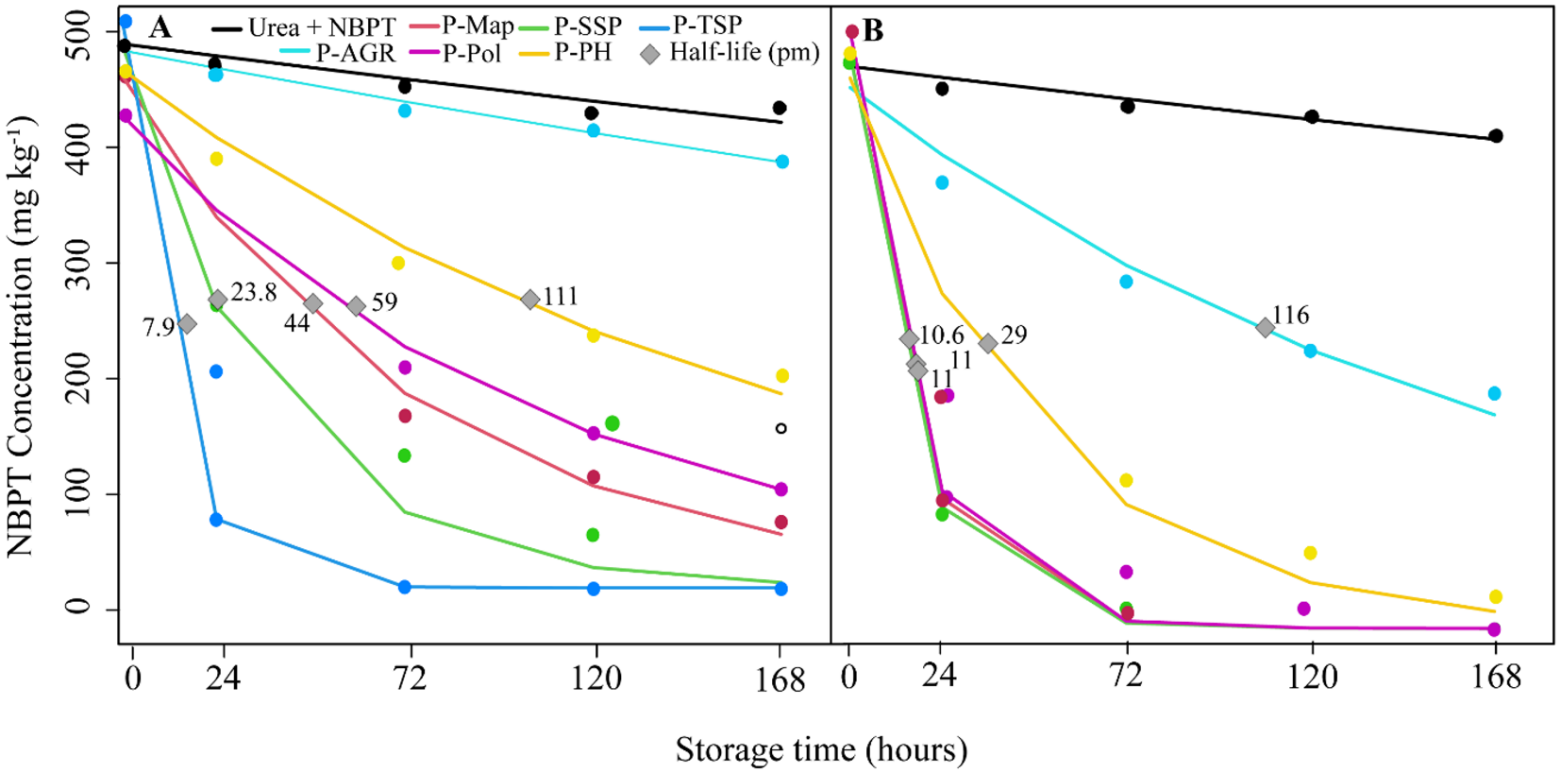
Variation of NBPT concentration over the storage period for conventional phosphated fertilizers and associated technologies at the ratios of 30 PF:70 NF (**A**) and 70 PF:30 NF (**B**), recorded at intervals of 0, 24, 72, 120, and 168 hours of storage.

Among the technologies for phosphate fertilizers (Agrocote—P_AGR_, Phusion—P_PH_, and Policote—P_POL_) in mixture with urea + NBPT (UR_NBPT_), the best compatibility was observed between UR_NBPT_ and P-_AGR_ fertilizer, mainly in the 30% P proportion. Regardless of the presence or absence of the technology in phosphate fertilizers, the degradation of the NBPT occurs over the storage time, reaching a concentration below 20% in up to 168 h (Fig. 1).

For the nitrogen fertilizer (NF) UR_NBPT_ and phosphate fertilizer (PF) mixture, the concentration of NBPT was linearly reduced according to the storage time. On average, the conventional fertilizers showed the NBPT degradation of 86% in the first 24 h for the mixture 70 PF:30 NF. In this group, the highest degradation in 24 h occurred with the Triple Superphosphate—P_SSP_ (100%) and the lowest with Monoammonium Phosphate—P_MAP_ (78%). The same fertilizers in the 30 PF:70 NF ratio showed a mean degradation of 66% in the first 24 h, with maximum degradation in Triple Superphosphate—P_TSP_ (87%) and minimum in MAP (50%).

The degradation of the NBPT mixed with phosphate fertilizers with associated technologies was, on average, 53.7% for the 70 PF:30 NF ratio and 24.3% for the 30 PF:70 NF ratio. The highest degradation in the first 24 h occurred with the P_POL_ fertilizer, both in the 70 PF:30 NF ratio (78.7%) and the 30 PF:70 NF (47.7%). The lowest NBPT degradation in both ratios occurred with the P_AGR_ fertilizer (21.5% and 4.3%, for 70 PF:30 NF and 30 PF:70 NF, respectively).

### Free-acidity of the phosphate fertilizers

The highest acidity was found in the conventional fertilizers (1.1, 0.39, and 0.29% for P_TSP_, P_SSP_, and P_MAP_, respectively). For the fertilizers with associated technologies, the free-acidity was 0.24, 0.15, and 0.14%, for P_AGR_, P_PH_, and P_POL_, respectively.

### NBPT degradation in urease inhibitors formulations during storage

**Figure 2:**
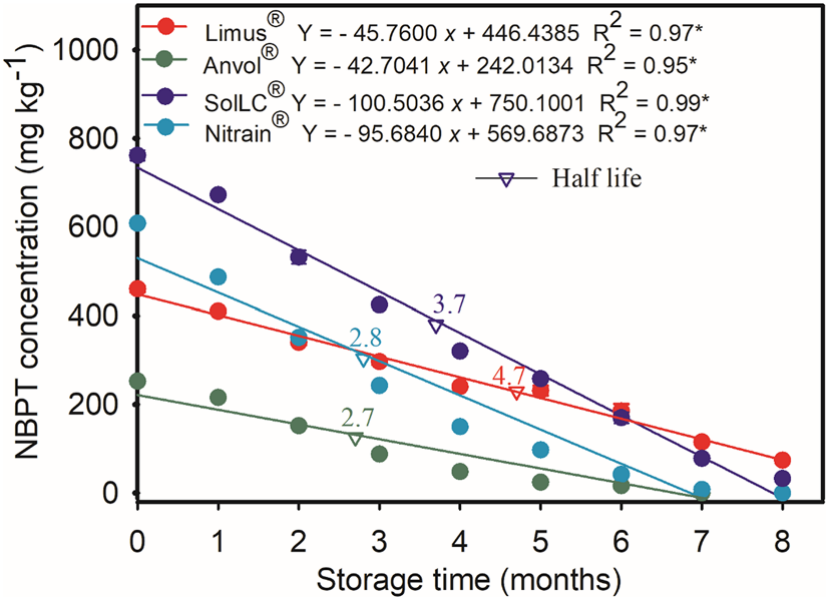
Concentration and residual NBPT in urea after 8 months of storage.

The urease inhibitors formulations showed a reduction in the concentration of NBPT during the 8 months of storage (Fig. 2). The values (in mg NBPT kg^−1^) found, respectively, before and after 8 months of storage were: 250 and 0 in Anvol; 460 and 115 in Limus; 600 and 7.5 on Nitrain; and 760 and 78 in SolLC, which is equivalent to reductions of about 250, 4, 80 and 9.7 times the initial concentration.

The daily rates of degradation of this same sequence of treatments were: 1.10, 1.89, 2.39, and 3.40 mg kg^−1^. It was not possible to detect the NBPT called Duromide (present in Anvol) in liquid chromatography, as well as the quantification of NPPT in Limus.

In this study, the half-life of the technologies was based on the proportion of NBPT applied during the preparation of the solvents following the instructions of the manufacturer. Thus, the half-lives for each technology were 4.7, 3.7, 2.8, and 2.7 months, for Limus, SolLC, Nitrain, and Anvol, respectively. We can conclude that the SolLC and Limus formulations demonstrated greater stability over the storage period.

### Urease inhibition technologies and their relationships with the urease activity in soil and ammonia volatilization

#### Edaphoclimatic conditions during the experiment

**Figure 3:**
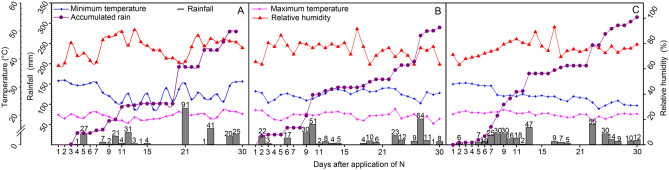
Climatic data of the experimental areas in the municipalities of Lavras (**A**), Ingaí (**B**), and Luminárias (**C**), during 30 days after the application of N to the soil.

The volumes of accumulated precipitation 7 days after nitrogen cover application (DAA) were 28, 42, and 48 mm for the localities of Lavras, Ingaí, and Luminárias (Fig. 3; Table S1). There was no precipitation on the first DAA. Only in Ingaí the precipitation in the first 4 days was higher to 6 mm (22 and 3 mm on days 2 and 3, respectively). The temperature and air humidity in the first four DAA reached the extremes of 32.4 °C (Lavras) and 80.5% (Lavras and Ingaí). High volumes of precipitation were associated with high temperatures (≥ 26 °C) between days 5 and 7, reaching 27.6, 17, and 42 mm in Lavras, Ingaí, and Luminárias, respectively.

The soils of the experimental areas of Lavras, Ingaí, and Luminárias showed the following initial contents: organic matter—4.2, 3.9, and 3.6% in the 0–5 cm layer and 3.4, 3.5, and 3.2% at 0–20 cm; N mineral stock—75.8, 55.6, and 49.4 kg ha^−1^ (0–5 cm) and 1764, 580, and 278 kg ha^−1^ (0–20 cm) (Tables S2 and S3).

#### Urease activity and ammonia volatilization

There were significant differences for the urease activity and daily volatilization regarding the sources of N. Under field conditions, each inhibition technology reduced urease activity in the soil, and lowered the intensity of ammonia emission peaks compared to granulated urea without NBPT (U_GRAN_) between 50 and 62%.

**Figure 4:**
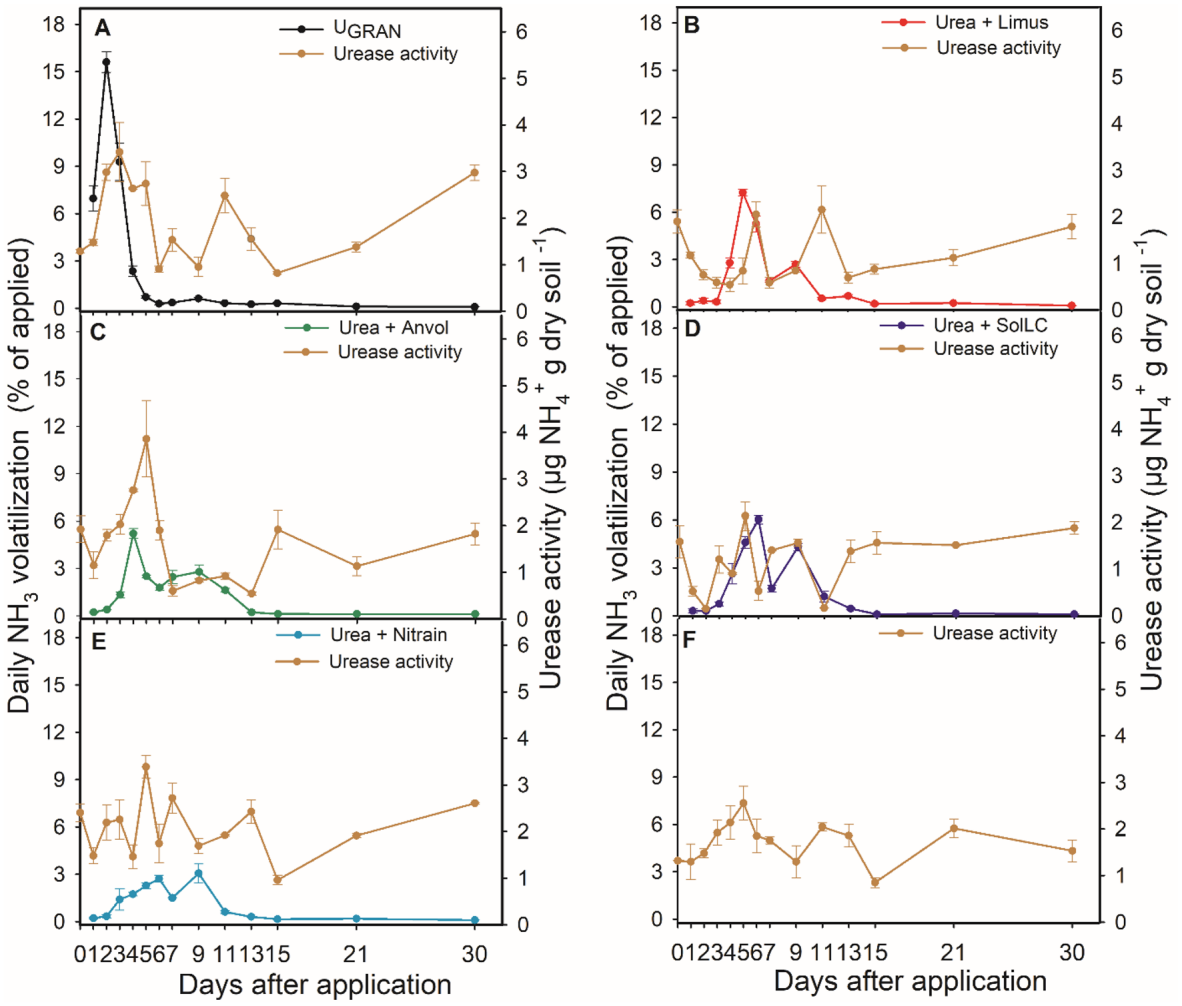
Volatilization of ammonia and soil urease activity 30 days after the application of N fertilizer technologies in Lavras, Minas Gerais, Brazil. Vertical bars indicate the standard error of the mean (n = 3).

In Lavras, the initial urease activity (day 0), before the N fertilization, was 1.73 µg NH_4_^+^ g of dry soil h^−1^. Even on the first day after fertilization, the urease activity remained constant in all treatments (Table S4). However, the NH_3_ losses were high, accounting 7% for U_GRAN_ (Table S5) on the first day and expressive losses up to the fourth day. The maximum loss occurred in 1.73 days (23.4 kg ha^−1^), where the urease activity was the highest among the treatments (approximately 3 µg NH_4_^+^ g of dry soil h^−1^) (Tables S3 and S5; Fig. 4). The daily losses in the plots that received urea treated with the technologies were below 1.5% of the N applied up to the 3 DAA. In this case, the urease activity was approximately 2 times higher for U_GRAN_. All formulations had maximum losses around the fifth DAA (7.5, 9.4, 3.8, and 4.8 kg ha^−1^ of NH_3_, for SolLC, Limus, Nitrain e Anvol, respectively) (Table S6). These losses coincide exactly with the highest values of urease activity where the urea treated with NBPT was applied and are mostly higher than U_GRAN_ and the control (Table S4). In this area, the urease inhibitors were responsible to reduce the maximum daily losses-MDL in 3.8, 3.4, 3.8, and 3.4 days for SolLC, Limus, Nitrain e Anvol (Table S6; Fig. 4).

**Figure 5:**
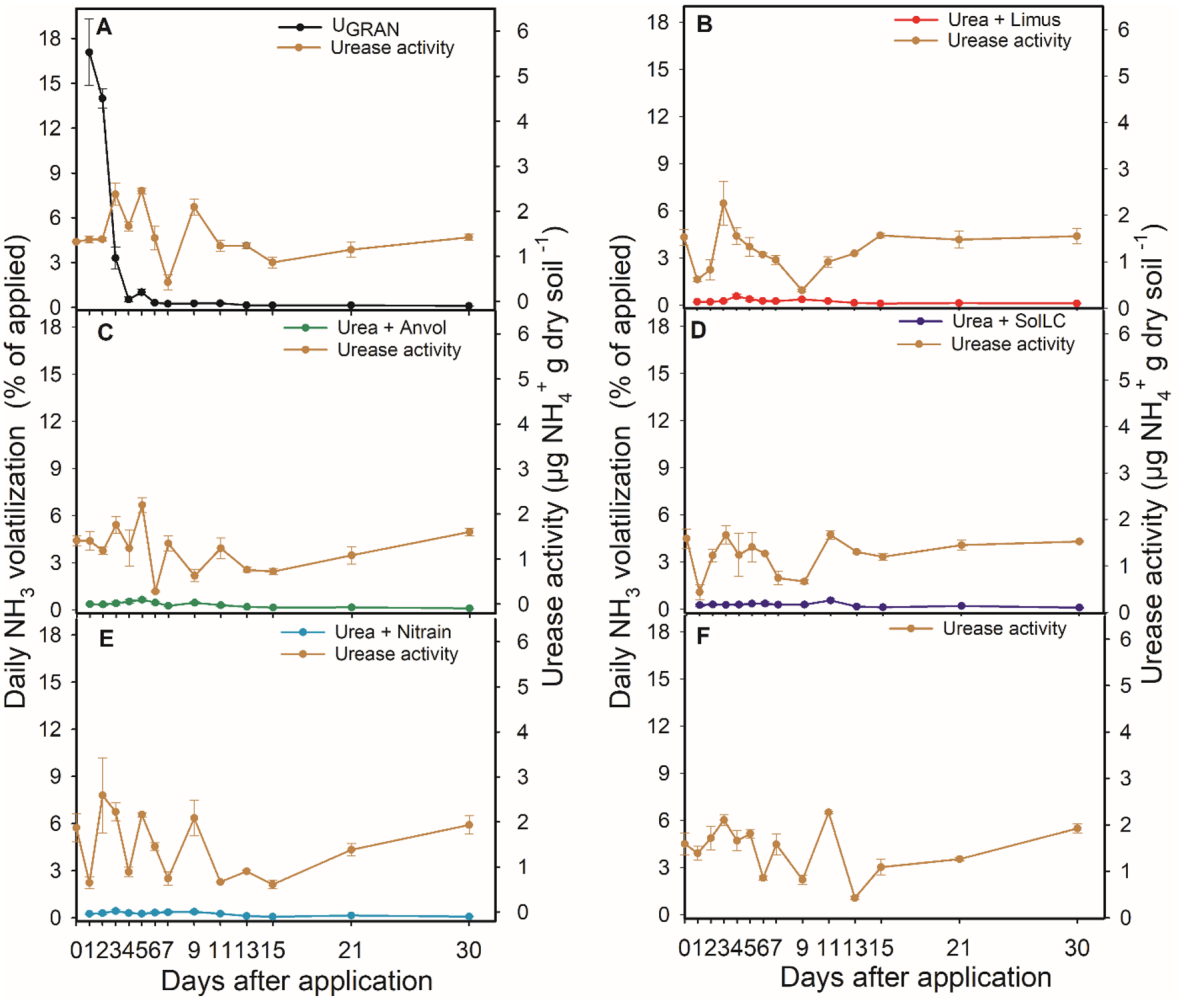
Volatilization of ammonia and soil urease activity 30 days after the application of N fertilizer technologies in Ingaí, Minas Gerais, Brazil. Vertical bars indicate the standard error of the mean (n = 3).

In Ingaí, the pattern was different from the other areas. The ammonia loss with U_GRAN_ (23 kg ha^−1^ of NH_3_) was maximum on the first day after fertilization, also coinciding with the highest activity of urease (1.37 µg NH_4_^+^ g of dry soil h^−1^), while on the other treatments the average urease activity was 0.77 µg NH_4_^+^ g of dry soil h^−1^ (Tables S3 and S6). The treatments that contained urease inhibitors had MDL between days 5 and 6 after fertilization, in particular for SolLC technology. However, the losses were inferior to 1 kg ha^−1^, while the urease activity was less than 0.5 µg NH_4_^+^ g of dry soil h^−1^ for most of the evaluated days. The formulations with urease inhibitors delayed the MDL by 3 to 5 days (Table S6; Fig. 5).

**Figure 6:**
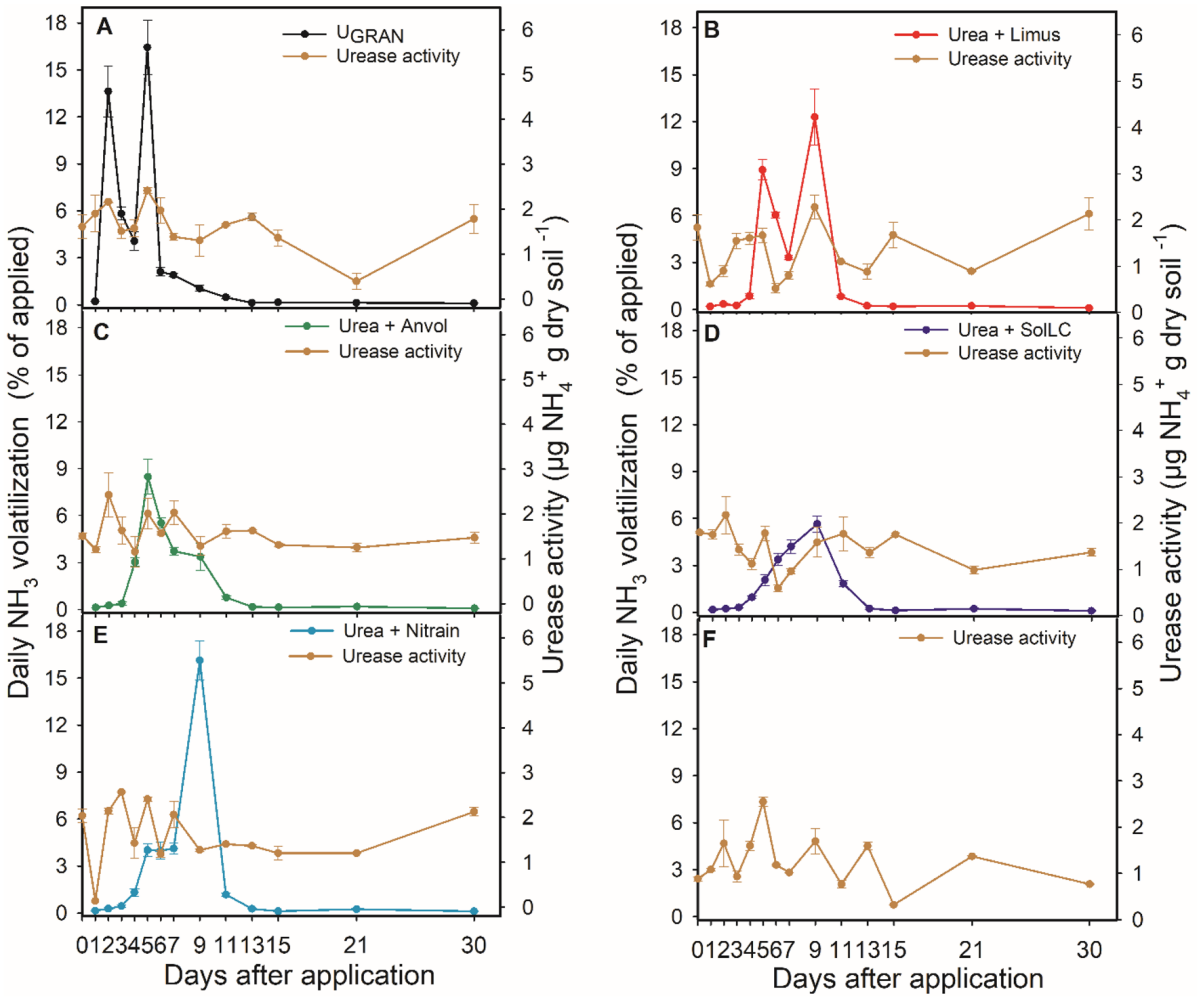
Volatilization of ammonia and soil urease activity 30 days after the application of N fertilizer technologies in Luminárias, Minas Gerais, Brazil. Vertical bars indicate the standard error of the mean (n = 3).

In Luminárias, U_GRAN_ showed a maximum loss in 3.4 days (15.5 kg ha^−1^), although not associated with the highest urease activity. The urease activity in this area did not have expressive differences among treatments, but it was higher where U_GRAN_ was applied and in the treatment without N application (Table S4; Fig. 6). However, we observed an increase in the urease activity in the fifth day in treatments with inhibitors, corresponding to 1.96 µg NH_4_^+^ g of dry soil h^−1^, in average, thus configuring the highest average activity up to the fifth day. This increase was associated with the maximum loss on treatments with inhibitors between days 5 and 6 (Table S6; Fig. 6). The losses accounted for 6, 11, 11, and 11 kg ha^−1^, for SolLC, Limus, Nitrain e Anvol, respectively. Reductions of 1.8 to 3.5 days in the MDL in this area were observed in relation to U_GRAN_.

**Figure 7:**
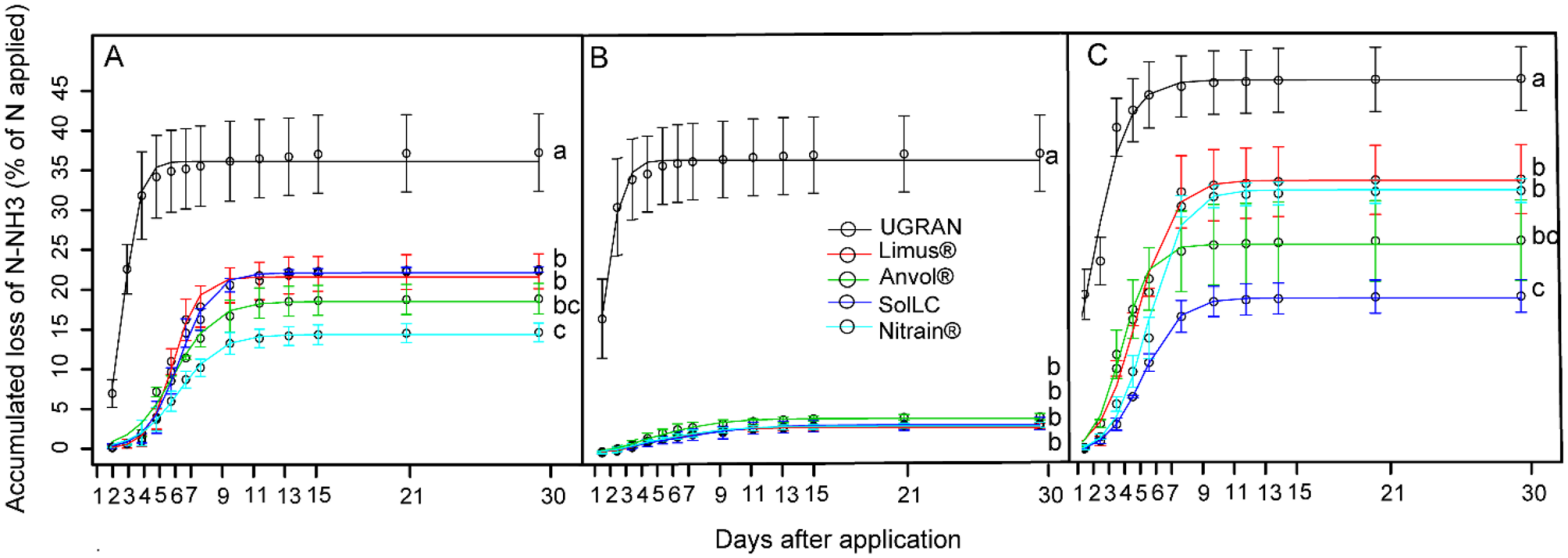
Accumulated losses of NH_3_ by volatilization 30 days after the application of N fertilizers in Lavras (**A**), Ingaí (**B**), and Luminárias (**C**). Means followed by the same letter do not differ according to Tukey’s test (P < 0.05). Vertical bars indicate the standard error of the mean (n = 3).

Considering the application to the soil of 150 kg N ha^−1^, NH_3_ losses ranged between 3% (4.5 kg ha^−1^) and 46% (67.5 kg ha^−1^). For the experiment conducted in Lavras, the highest accumulated loss accounted for 36% (54 kg ha^−1^) using U_GRAN_, while the lowest losses were 14.4% for (21.6 kg ha^−1^) for Nitrain and 18.5% (27.7 kg ha^−1^) for Anvol (Fig. 7A). The other treatments in this region did not differ and, in average, accounted for 21.8% (32.7 kg ha^−1^) of ammonia loss. In Ingaí, the highest losses were also with U_GRAN_ (36.7%, 55 kg ha^−1^), while there was no difference among the other treatments (3.47% on average; 5.2 kg ha^−1^) (Fig. 7B).

In Luminárias, we observed high NH_3_ losses in all treatments, accounting for 31.4%; 47 kg ha^−1^, on average (Fig. 7C). In this area, U_GRAN_ showed a loss of 46% of the N applied (69 kg ha^−1^), followed by Limus (33.6%; 50.4 kg ha^−1^) and Nitrain (32.5%; 48.7 kg ha^−1^). Anvol showed intermediary loss (25.8%; 38.7 kg ha^−1^). SolLC showed the lowest accumulated loss of 19% (28.5 kg ha^−1^).

#### Agronomical results in the experiment with maize

**Figure 8:**
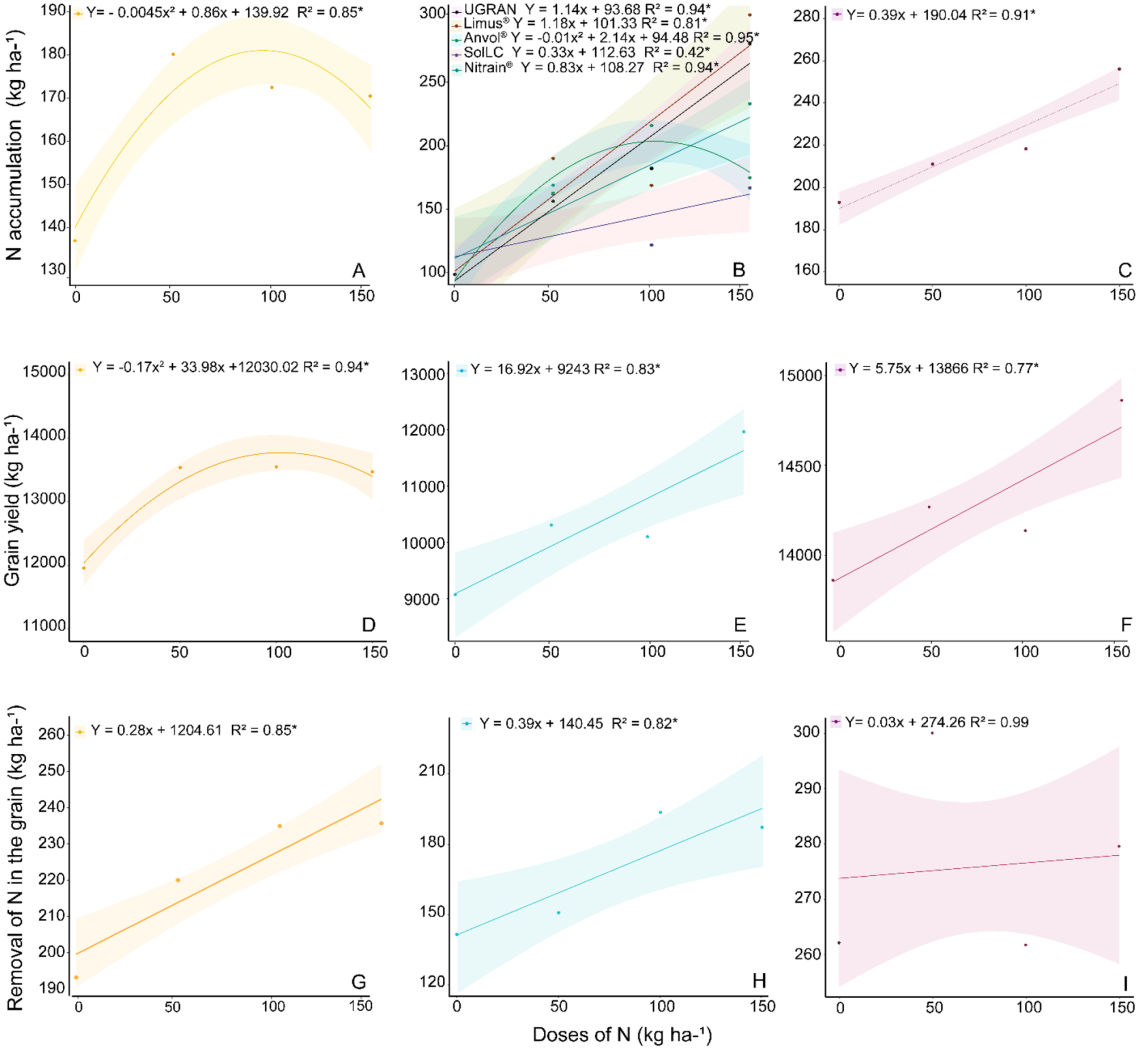
Nitrogen accumulation in the shoots during the flowering stage for the different rates of N applied as cover fertilization in maize cultivated in the municipalities of Lavras (**A**), Ingaí (**B**), and Luminárias (**C**), Minas Gerais, Brazil. Yield as a response to the rates of applied N in Lavras (**D**), Ingaí (**E**), and Luminárias (**F**). N removal by the grains in Lavras (**G**), Ingaí (**H**), and Luminárias (**I**). The shaded area represents the confidence interval (n = 3).

There was an influence of the rate of N on all agronomical variables (Fig. 8). In the area of Ingaí, however, the effects of the treatments over the NAS varied according to the treatment.

Yields higher than 9200 kg ha^−1^ of grains were achieved even in the absence of N fertilization. In the treatment that did not receive N, the average yield for the areas of Lavras, Ingaí, and Luminárias were 12,030, 9243, and 13,867 kg ha^−1^, respectively, when the N in the plants varied from 90 to 190 kg ha^−1^. In addition, increasing rates of N linearly increased the NAS up to 249 kg ha^−1^ and yield to 14,808 kg ha^−1^ in Luminárias; yield to 11,781 kg ha^−1^ and N removal to 203 kg ha^−1^ in Ingaí; and N removal to 247 kg ha^−1^ in Lavras (Fig. 8).

In Lavras, the NAS decreased to less than 181 kg ha^−1^ when the rate of N exceeded 95.6 kg ha^−1^. Despite the N fertilization, the highest yield in this area (13,772 kg ha^−1^ of grains) was achieved with the N rate of 100 kg ha^−1^. Above this rate, results were gradually reduced. At a similar rate (100 kg ha^−1^), the application of Anvol reduced the NAS in Ingaí, while for the other technologies, each kilogram of N provided by U_GRAN_, SolLC, Limus e Nitrain increased NAS by 1.14, 0.32, 1.18, and 0.83 kg ha^−1^, respectively (Fig. 8).

## Discussion

### Storage compatibility of mixtures between urea treated with NBPT and phosphate fertilizers

The results showed in this work confirm the significant impact on the degradation of the urea treated with NBPT due to the release of phosphoric acid during storage time by phosphate fertilizers^17^. The mixtures in this work were not based on the P_2_O_5_ concentration but rather on proportion of fertilizers. We observed that especially in the proportion 70% P (30 NF:70 PF), the reduction on the concentration of NBPT was more pronounced. It is known that such phosphate fertilizers are more acidic due to the manufacturing process^25^. Consequently, the higher free acidity, especially from the triple superphosphate (1.10%), drastically accelerated the degradation of the NBPT during the storage.

The coating of the Agrocote granules probably reduced the contact between MAP and urea treated with NBPT. Regarding the other fertilizers (Policote and Phusion), the granulation technologies probably did not coat the entire granules, thus leading to a higher degradation of the NBPT when compared to Agrocote. As there was less acidity in the phosphate fertilizers tech associated with technologies compared to conventional fertilizers^26^, there was a higher concentration of the inhibitor at the end of the storage.

### NBPT degradation in urease inhibitors formulations during storage

The decrease in the concentration of NBPT along 8 months of storage was verified for all the NBPT formulations. This pattern can be explained as a consequence of storage time and the storage temperature (25 °C, considered an average for tropical climates), confirming that the stability of the NBPT is dependent on both factors^27^. Watson et al.^27^ highlighted the influence of the conditions during the storage of urease inhibitors, which can cause future loss of efficacy in the field^17^. In addition, the authors emphasize that even with the application to the urea, the NBPT tends to degrade, limiting the lifespan of the additive in the fertilizer.

In addition to the temperature and storage time factors, we observed that the composition of the formulation also tends to be an important factor for the storage of the product before its application in the field.

Was observed that the stability of the fertilizers treated with NBPT formulations depends on the additive used. The positive results of Limus may be related beyond due to NBPT, by the higher concentration of the NPPT in the formulation, which is less soluble than the NBPT thus granting higher stability during storage^28^. Even at initial concentrations of NBPT lower than those of Nitrain, a better concentration of the Limus inhibitor was obtained at the end of the storage test (residual NBPT of approximately 40% at the end of the test).

In formulation Nitrain the associated additive did not promote efficient stabilization during the storage, reducing the residual concentration close to the concentration found in Anvol (< than 20%). NBPT concentrations close to what is usually commercialized before field application are important to improve the efficiency of the additive molecule in reducing NH_3_ losses when treated urea is applied in the field. Similarly, the proper concentration of the inhibitor is important to achieve a half-life of the fertilizer during storage. We observed this pattern for SolLC.

However, it is important to highlight that the variation in the half-life of the formulations from this study does not determine the efficacy of the inhibitor in the field. So far, we only discussed the reduction of the NBPT concentration in the urea after the storage time.

### Urease inhibitors formulations and their relationships with the urease activity in soil and ammonia volatilization

In this study, we observed significant impacts on N losses after the application of urea without the use of formulations capable of inhibiting soil urease activity. This probably occurred due to the rapid hydrolysis of urea favored by the wetter soil associated with high temperatures (especially in Lavras and Luminárias), which granted the highest urease activity^16,22,29,30^.

Considering the 150 kg N ha^−1^ supplied to the soil via cover fertilization, they were lost as NH_3_ at the end of the evaluation cycle: between 3.4 and 22% with SolLC; between 3 and 33.6% with Limus; between 3.2 and 32.5% with Nitrain; and between 4.2 and 25.8% with Anvol. Overall, the formulations were efficient to reduce NH_3_ losses in relation to U_GRAN_, mitigating losses between 50–62%.

This is explained by the fact that the active sites of the NBPT molecule act in blocking the sites of urease activity in the soil blocking, for a period, the hydrolytic action of urea, consequently, there is a reduction of N to the atmosphere through the use of this formulation^31^.

Studies report that the period of inhibition of urease activity by NBPT comprises from 3 to 14 days, depending on the concentration of the inhibitor and edaphoclimatic conditions^16,27,32–34^.

Nitrain formulation showed the best performance to reduce the N loss in Lavras. We did not find reports in the literature using this formulation in the field. The ability of Nitrain, associated with a biocatalyst provided a good assessment of its effectiveness in the field to reduce NH_3_ loss rates. This formulation reduced the NH_3_ losses by up to 59.6%, in relation to U_GRAN_ even when the climate conditions (humidity > 60% and average temperature > 20 °C) and urease activity were substantially favorable to volatilization during the first week after fertilization.

The occurrence of rains during the experiment in Ingaí was important to incorporate the urea into the soil, allowing greater reduction of NH_3_ losses by the formulations when compared to other experimental areas^27,35^. In this área, SolLC was the formulation more efficient in delaying the period of greatest loss in relation to urea. In Luminárias, the same technology exhibited a higher reduction in NH_3_ volatilization by up to 60% as well as to provide a better reduction in urease activity, compared to U_GRAN_. The results observed in both cases can be explained by the higher concentration of the inhibitor in the formulation, which provided better inhibition of the urease around the granules. When using high concentrations of NBPT (1500 and 2000 mg kg^−1^) the CDM can be reached around 6 days after application of N, however, this value is dependent on the environmental conditions during application^36^. Another aspect may be related to the rate of biodegradation of the inhibitor and its persistence in the soil, which was essential to prolong the day of maximum loss^16^.

The N accumulation in the maize was close (in Lavras) or higher (in Luminárias) to those required by the crop (180 to 200 kg ha^−1^ of N^37^). In Ingaí, in absence of the fertilization initial levels of N in the plant were lower, and therefore, the supply of formulations (except for Anvol) resulted in significant increases after application of doses of N.

The difference observed in the accumulation of N in the plant among the U_GRAN_ and formulations in Ingaí can be related to the intense rainfall that incorporated the urea into the soil immediately after the beginning of the NH_3_ volatilization and to the higher N need in this area.

In addition, the maize cultivated in Ingaí was responsive to the application of N rates increasing in up to 28% with the maximum dose (150 kg ha^−1^ of N). This response is explained by the lower availability of N in the soil. Conversely, the absence of responses of urea with or without the associated formulations in most agronomic evaluations in the areas of Lavras and Luminárias is given by the reserves of organic and mineral N in the soils by a no-tillage system consolidated for years. This became evident when the control (without N application) surpassed 2.8 and 3.2 times the mean yield (4214 kg ha^−1^) for the state of Minas Gerais (Brazil) for the crop season of 2020/2021, this increase represents 131 and 161 bags of grains per hectare in Lavras and Luminárias, respectively, only relying on the constructed and consolidated soil fertility of these areas. The application of nitrogen, regardless of the NBPT formulation used, together with the nitrogen reserves in the soil, resulted in the accumulation of sufficient nitrogen to drive increased productivity. In addition, it is important to note that nitrogen losses by volatilization did not have a direct impact on the agronomic results analyzed in this study. However, without replacing the extracted N from the soil, through the adequate management of N and the use of technologies, the reserves constructed through years can be reduced in the medium and long term. Therefore, it is important to use formulations to reduce N volatilization and that contributes to the replenishment of N in the soil of cultivated area.

### Future perspectives

In summary, our results indicate a reduction in the stability of NBPT when stored in a mixture with phosphate fertilizers, provided that these fertilizers have a good coating without causing any contact with the NBPT. However, even with the coating, it is important to store the fertilizer in optimal conditions for a time that does not reduce the efficiency of the NBPT after application in the field.

In addition, for a better choice of a formulation containing NBPT, we recommend the use of technologies that ensure the stability of the additive, or opt for a formulation that initially contains a higher concentration of NBPT. This will ensure that, when used by the producer, the formulation presents a minimum effective concentration to reduce nitrogen losses by volatilization.

In addition, our studies also indicate the contribution of different formulations of urease inhibitors to urea with the purpose of promoting the increase in the efficiency of the use of N as well as contributing to the longevity of the formulations during the storage and further stability in the field, although the performance is dependent on the bioedaphoclimatic conditions of the area.

We emphasize the importance of the half-life of the NBPT formulations during storage. This information should be provided to the consumer through the batch manufacturing information, so it would be known when the acquired technology will have its efficiency reduced by the degradation of the NBPT. With such information, consumers could opt for more efficient and stable technologies during storage.

## Materials and methods

### Storage compatibility of mixtures between urea treated with NBPT and phosphate fertilizers

Two essays were conducted to quantify the concentration of *N*-(*n*-butyl) thiophosphoric triamide (NBPT) in urea treated with NBPT after its storage with phosphate fertilizers. The essays differed in the proportion of N fertilizer (% NF) and phosphate fertilizer (%PF) in the mixture. In essay I, the proportion was 30 NF:70 PF (granulated urea + NBPT and phosphate fertilizer), and in essay II, a 70 NF:30 PF proportion was used. Both essays were set up in a completely randomized design, with six replications, and a (6 × 5) + 1 factorial scheme. The factorial corresponded to the mixture of urea + NBPT (UR_NBPT_) and six phosphate fertilizers (conventional: monoammonium phosphate, single superphosphate, triple superphosphate, and the technologies for phosphate fertilizers: Policote, Phusion, and Agrocote), and five storage times (0, 24, 72, 120, and 168 h), and additional treatment with only UR_NBPT_.

### Preparation of fertilizers

#### Urea treatment with urease inhibitors technologies

To standardize the urea granules, the samples were sieved to a diameter between 4 to 3.35 mm. Then, they were homogeneously split using a SONDATERRA fertilizer splitter. The technologies added to granulated urea (46% N) were provided by the manufacturers or developed by the Innovations for fertilizers Research Group. Except for the Anvol (ready-to-use formulation, commercially available), the entire mixing process with granulated urea was performed at the UFLA's Laboratory of Innovations for Fertilizers. The mixture was homogenized in a Super Bio Fast benchtop mixer for 5 min at 30 rpm.

Specifications on the studied fertilizers are described below.

**SolLC technology:** Prepared with a solvent developed by the InnovaFert-UFLA Research Group. After testing several mixtures of solvents, the complete dilution of the product was obtained in a single solvent, herein named SolLC. A known NBPT (P.A.) amount was added to SolLC solvent (used at approximately 20% concentration but can be raised to 40% without crystal formation). The mixture was then subjected to specific laboratory processes, which ensured the complete dilution of the powder. After this process, the product was colored (1% by weight of dye) and applied to urea at a rate of 3.04 kg t^−1^.

**Limus technology:** Developed and patented by the BASF company. It contains the urease inhibitors NBPT (75%) and *N*-(*n*-propyl)-thiophosphoric-triamide (NPPT, 25%) (3:1 ratio). Formulation applied at a rate of 2 kg t^−1^.

**Nitrain technology:** According to the manufacturer, Nitrain's formulation ensures better nitrogen utilization efficiency, boosting crop growth. This formulation was applied at a rate of 1.9 kg t^−1^.

**Anvol technology:** Developed and patented by Koch Agronomic Services, considered an industrial standard treatment for this study. The Anvol additive consists of 43% NBPT, of which 27% refer to Duromide [NBPT adduct and main active ingredient of the product and 16% to free NBPT, which would be an “immediately available” fraction of the additive^18^. The formulation was applied at a rate of 1.5 kg t^−1^.

#### Phosphate fertilizers

Phosphate fertilizers (PF) were used in this study to compare their effects on the degradation of NBPT in urea after storage over time. The phosphate fertilizers used in the present study were purchased from a fertilizer store. The same procedures of item 1.1.2 were applied to standardize the diameter of the fertilizer granules. The conventional fertilizers used were (1) Monoammonium Phosphate (PMAP), (2) Single Superphosphate (PSSP) and (3) Triple Superphosphate (PTSP). Fertilizers containing some type of associated technology were (4) Policote Phos (PPOL): MAP fertilizer with anionic polymers (Policote), containing 10% N and 49% P2O536, (5) Phusion (PPH): Fertilizer with 40% P_2_O_5_, macro and micronutrients, and incorporation of fulvic and humic acids^36^, (6) Agrocote E-max (PAGR): MAP fertilizer coated with polyurethane. It is coated with a polymer named E-max Release Technology™, consisting of 9% N and 47% P2O5^36^.

#### UR_NBPT_ mixture with phosphate fertilizers

The mixtures between phosphate fertilizers and granulated urea treated with NBPT were performed as follows: Five grams of each proportion of the mixtures of URNBPT and phosphate fertilizers (70:30 and 30:70) were placed in glass vials (25 mL). The vials were closed, manually shaken, and stored in a BOD chamber at 25 °C. After 0, 24, 72, 120, and 168 h of storage, the vials were removed from the chamber. Then, urea granules and phosphate fertilizers were separated (Fig. S1).

#### UR_NBPT_ dissolution after separation of phosphate fertilizers

After separating the granules, UR_NBPT_ was dissolved in ultrapure water, considering the same proportion used for phosphate fertilizers. An aliquot of 1 mL of this solution was collected and stored in an amber vial. Then, the samples were stored in the freezer. Such procedure aimed to preserve the NBPT molecule in the solution until its quantification.

#### NBPT quantification in urea after storage

The quantification of the NBPT concentration was performed by liquid chromatography (HPLC), in an Agilent device model HP1100, with a diode array detector. The quantification followed the method described by the European Committee for Standardization^38^.

#### Calculation of NBPT longevity (half-life)

By definition, the half-life corresponds to the time required to reduce a certain concentration to 50% of the initial value^27^. This parameter was equalized between the treatments, isolating the time variable. Further descriptions of the method will be described in the statistical analysis section.

#### Determination of the free-acidity in phosphate fertilizers

Free-acidity was determined by titration, as described in ABNT NBR 5774:2010^39^. Two grams of PF were extracted with neutralized acetone and then titrated with a standardized sodium hydroxide solution (0.1 mol L^−1^), using alizarin as an indicator. The result was expressed as a percentage (PF weight) of phosphoric acid.

#### NBPT degradation of urease inhibitors formulations as a function of storage time

The NBPT degradation of additive formulations containing urease inhibitors mixed with granulated urea was monitored during 8 months of storage. Granulated urea (U_GRAN_ without NBPT) was treated with four NBPT-containing technologies: SolLC; Limus, Nitrain, and Anvol.

The resulting mixtures were stored in sealed plastic bags, in triplicate, under controlled conditions of temperature (25 °C) and relative humidity (76%). The initial and residual NBPT concentration (every 30 days of storage) of each technology was determined by liquid chromatography (HPLC). The half-life calculation also followed the procedures described earlier.

#### Efficiency of the urease inhibition technologies under field conditions

Three field experiments were conducted to evaluate the efficiency of urease inhibitor formulations for urea and to verify their effects on urease activity, ammonia volatilization, plant nutrition, and maize yield.

#### Characterization of the experimental areas

The experiments were carried out simultaneously in different areas of Minas Gerais state, Brazil. To represent the geographical distribution of the study areas a map was generated using ArcGis software version 11.0 (Fig. S2). Experiments I and II were conducted in a Latossolo Vermelho (Oxisols, USDA) in Lavras (21°16′00″ S; 44°57′27″ W) and Ingaí (21°22′08″ S; 44°53′23″ W). As for experiment III, it was conducted in a Cambissolo Háplico (Inceptsols, USDA) in Luminárias (21°29′59″ S; 45°00′26″ W). Tables S2 and S3 list the characterization of the three soils. These locations have a Cwa climate type, subtropical with dry winters and rainy summers, with a mean temperature between 19.6 and 21 °C. Lavras and Ingaí are cultivated under a no-tillage system in consolidation. In turn, the soil in Luminárias was cultivated with eucalyptus, and was recently converted to a grain production area. The succession of soybean/corn or soybean/wheat is adopted in all areas. All areas were cultivated with soybean prior to the installation of the experiments.

#### Experimental design

The experiments were set in a randomized block design, with three replications, in a factorial scheme 5 × 4. The factorial consisted of five treatments [untreated, granulated urea without NBPT (U_GRAN_), and urea treated with the urease inhibition technologies SolLC, Limus, Nitrain, and Anvol] and four N rates (0, 50, 100, and 150 kg ha^−1^).

Each experimental unit consisted of five sowing rows with 5 m-length (10 m^2^). The three central rows were considered the useful plot area (4.5 m^2^, after discarding one meter at each ending).

#### Maize sowing and management practices during cultivation

Maize was sown in the first season of 2020 (November), using the AG8070 PRO3 hybrid. The sowing spacing was 0.5 m between rows, totaling 70,000 plants ha^−1^. Potassium fertilization was conducted 15 days before sowing using 90 kg ha^−1^ of K_2_O as potassium chloride. At sowing, 300 kg ha^−1^ of 13-33-00 were applied. Nitrogen fertilization with urea and urease inhibitor formulations was performed in one topdressing application, after the development of the third pair of maize leaves (V3 vegetative stage, approximately 25 days after emergence). The fertilizers were manually applied at the 50, 100, and 150 kg N ha^−1^ rates.

All experimental areas received the same management practices. At 45 days after sowing (DAS), fungicide based on trifloxystrobin (100 g L^−1^) + tebuconazole (200 g L^−1^), and acaricide based on mancozeb at doses of 0.6 L ha^−1^ and 2 kg ha^−1^, respectively, were applied. At 60 DAS, the second fungicide application was performed, with a product based on picoxystrobin (200 g L^−1^) + cyproconazole (80 g L^−1^) at a dose of 400 mL ha^−1^. Applications were performed on the same day in the three experimental areas.

#### Monitoring of weather conditions

Weather information from Lavras was provided by the National Institute of Meteorology—INMET. Data from Ingaí and Luminárias were collected at private climatological stations installed in each experimental area.

#### Determination of urease activity, soil pH, and ammonia volatilization

Soil samples were collected at the 0–2 cm depth in the fertilization line, with a metal spatula. Samplings occurred in the 1st, 2nd, 3rd, 4th, 5th, 6th, 7th, 9th, 11th, 13th, 15th, 21st, and 30th days after the N fertilization in the plots that received 150 kg N ha^−1^, and in the control without N.

The soil was sieved to 2 mm to standardize the samples and remove plant remains. Then, soil samples were stored in properly identified plastic bags and kept under refrigeration at 4 °C for up to 2 weeks before the analysis.

**Urease activity:** The quantification of urease activity in soil is based on the determination of the ammonia released after incubating a soil sample in urea solution^40^. It was determined using 5.0 g of soil, 9 mL of Tris THAM buffer (pH 9), and 1 mL of urea solution (0.2 M), incubated at 37 °C for 2 h. Then, 35 mL of KCl–Ag_2_SO_4_ solution (2.5 M, 100 mg L^−1^) were added to stop the reaction. The solution was stirred and left standing for 5 min at room temperature. The volume was completed to 50 mL with KCl and Ag_2_SO_4_, followed by stirring. Then, 20 mL aliquots of the supernatant were transferred to digestion tubes, which received 0.2 g of MgO and were taken to the distillation process^41^. Finally, the solution was titrated with H_2_SO_4_ solution (0.005 M), using a boric acid solution, methyl red, and bromocresol green as indicators.

**Soil pH:** Determined using a combined electrode inserted in a soil suspension and CaCl_2_ solution (1:2.5 v/v), according to the methodology proposed by Embrapa^42^.

**Ammonia (NH**_**3**_**) volatilization:** The NH_3_ losses by volatilization were quantified using the PVC semi-open collector method^43^. Three PVC tube bases (20 cm in diameter and 20 cm in height) were installed in each plot, at 10 cm from the maize sowing row. The respective treatments were applied inside the PVC bases, proportionally to the base area, without mechanical incorporation or by irrigation. Soon after, a collector made of PVC (20 cm in diameter and 50 cm in height) was installed on the first base. Two sponges (0.02 g cm^−3^ density) embedded with phosphoric acid (60 mL L^−1^) and glycerin (50 mL L^−1^) were placed in each collector. The sponge in the upper part of the collector aimed to prevent a possible contamination of the lower sponge (used to capture the volatilized ammonia).

At each sampling, the sponges were collected and sent to NH_3_ quantification, and then replaced by new ones. The chambers were rotated within the three bases of the respective treatments. Such practice aimed to reduce the spatial variability of ammonia emission and the formation of a microclimate inside the collectors^44^. NH_3_ losses were daily quantified in the first 7 days after fertilization with N-based technologies (DAFNT), when the NH_3_ losses are often higher^22^. After that, on alternate days and pre-defined dates (coinciding with the soil sampling for quantification of urease activity).

The sponge solution was extracted by filtration using a Büchner funnel and a vacuum pump, after five sequential washes with 80 mL of deionized water. Aliquots of 20 mL were taken from the obtained extract to determine the N content by distillation). The result was converted to the NH_3_ losses (%) per hectare. The accumulated losses were calculated from the sum of days 1 and 2, which was added to the losses of day 3 and so on, until day 30^41^.

#### N accumulation in plant shoot

At the flowering stage, three maize plants were collected per plot (one plant in each of the three central rows). Then, they were dried at 65 °C until constant weight, weighed, and ground. The material was newly ground in a Willey mill for better homogenization. In the laboratory, the N content in plant material was determined after sulfuric digestion. The standard reference material (NIST—1573A—Tomato Leaves) with a concentration of 29.2 g N kg^−1^ was used to evaluate the accuracy of the N determination method. The results of NIST analysis showed 100% recovery.

The N accumulation in maize shoot (NAS) was obtained by multiplying the N content by the mass of the three plants. Then, the obtained value was converted to kg ha^−1^.

#### Grain yield

Maize cobs of fifteen plants were harvested from the useful area of all plots to estimate grain yield. The cobs were threshed, the grains were weighed, and moisture was quantified. Maize yield was then estimated using the number of plants per hectare. The sample moisture was used to calculate the correction to a moisture of 130 g of water kg^−1^.

#### N removal by the grains

After yield determination, grain subsamples were oven dried at 65 °C until constant weight. They were ground in a Willey mill, and their N contents were determined by the Kjeldahl method with NIST validation (1573A—Tomato Leaves). The N removal by the grain was given by multiplying the N content by maize yield, and then the values were converted to kg ha^−1^.

We confirm that the plant study complies with relevant institutional, national, and international guidelines and legislation. The seeds used were duly registered in the Brazilian Ministry of Agriculture, Livestock and Supply (MAPA) and were acquired in local trade authorized by the competent body RENASEM (National Registry of Seeds and Seedlings).

### Statistical analyses

The NBPT degradation was calculated using a nonlinear regression model. This model is indicated to describe a decay pattern according to Eq. (1):

1 $${\text{Y}} = \alpha *{\text{e}} - {\text{k}}*{\text{t}} + \epsilon 1,$$

in which *Y* is the NBPT concentration (mg kg^−1^); *t* represents the storage time (h) of the fertilizer granules; *α* corresponds to the initial condition of the plot, that is, the estimate of 100% of the applied amount of NBPT; *k* is the value indicating the NBPT degradation, which refers to the variation of NBPT losses over time; $\epsilon 1$ is the random error associated with the i-th observation.

The half-life was estimated based on the Henderson model, in which Eq. (1) was equaled to the half-life of urea treated with NBPT, and the time variable was isolated. Half-life was estimated according to equation:

$$t=\frac{\frac{{\text{ln}}\left(HL\right)}{\alpha }}{-k}$$

in which HL represent the NBPT half-life. The statistical analyses were performed using the R 3.3.1 software^45^.

Analysis of variance was performed for field data. When the effects of technologies were significant, they were compared using the Tukey test (P < 0.05). Linear or quadratic regression models were adjusted to evaluate the effect of N rates on N accumulation in the plant, grain yield, and N removal by the grains. All analyses were performed using the statistical program SISVAR version 5.7 and software R 3.3.1^45^.

The accumulated ammonia losses were subjected to nonlinear regression analysis using the logistic model *Y*_*i*_ = [*α*/{*1* + *e*^*k*^ (*b* *−* *daa*_*i*_)}] + *E*_*i*_), in which: *Yi* is the i-th observation of the accumulated NH_3_ loss (%), and i = 1.2…n; *daa*_*i*_ is the i-th day after application; *α* is the asymptotic value that can be interpreted as the maximum accumulated value of NH_3_ loss; *b* is the abscissa of the inflection point and indicates the day when the maximum volatilization loss occurs; *k* is the value that indicates the precocity index [the higher the *k* value, less time will be needed to reach the maximum accumulated loss value (*α*)]; *E*_*i*_ is the random error associated with the i-th observation, which is assumed to be independently and identically distributed according to a norm of zero mean and constant variance, E ~ N (0, I σ2). To estimate the maximum daily loss, that is, to determine the curve inflection point, the equation was used: *MDL* = (*k* × *α*)/4.

### Supplementary Information

Supplementary Information.

## Data Availability

The datasets used and/or analysed during the current study available from the corresponding author on reasonable request.

## References

[1] IFA. Estimating and Reporting Fertilizer-Related Greenhouse Gas Emissions: Linking Fertilizer Best Management Practices with national climate change mitigation targets 1–12 (2018).

[2] Zhang X (2015). Managing nitrogen for sustainable development. Nature.

[3] Cantarella H, Novais RF (2007). Fertilidade do solo—Nitrogênio. Fertilidade do solo.

[4] Omara P, Aula L, Oyebiyi F, Raun WR (2019). World cereal nitrogen use efficiency trends: Review and current knowledge. Agrosyst. Geosci. Environ..

[5] Timilsena YP (2015). Enhanced efficiency fertilisers: A review of formulation and nutrient release patterns. J. Sci. Food Agric..

[6] Trenkel ME (2010). Slow-and Controlled-Release and Stabilized Fertilizers: An Option for Enhancing Nutrient Use Efficiency in Agriculture.

[7] Chojnacka K, Moustakas K, Witek-Krowiak A (2020). Bio-based fertilizers: A practical approach towards circular economy. Bioresour. Technol..

[8] Pan B, Lam SK, Mosier A, Luo Y, Chen D (2016). Ammonia volatilization from synthetic fertilizers and its mitigation strategies: A global synthesis. Agric. Ecosyst. Environ..

[9] Department for Environment, Food and Rural Affairs. *Committed Clean Air Zone Impact Assessment* 1–100 (2016).

[10] Chien SH, Prochnow LI, Cantarella H (2009). Chapter 8 Recent Developments of Fertilizer Production and Use to Improve Nutrient Efficiency and Minimize Environmental Impacts. Advances in Agronomy.

[11] Lawrencia D (2021). Controlled release fertilizers: A review on coating materials and mechanism of release. Plants.

[12] Kafarski P, Talma M (2018). Recent advances in design of new urease inhibitors: A review. J. Adv. Res..

[13] Cameron KC, Di HJ, Moir JL (2013). Nitrogen losses from the soil/plant system: A review. Ann. Appl. Biol..

[14] Dimkpa CO, Fugice J, Singh U, Lewis TD (2020). Development of fertilizers for enhanced nitrogen use efficiency—Trends and perspectives. Sci. Total Environ..

[15] Keshavarz Afshar R, Lin R, Mohammed YA, Chen C (2018). Agronomic effects of urease and nitrification inhibitors on ammonia volatilization and nitrogen utilization in a dryland farming system: Field and laboratory investigation. J. Clean. Prod..

[16] Klimczyk M, Siczek A, Schimmelpfennig L (2021). Improving the efficiency of urea-based fertilization leading to reduction in ammonia emission. Sci. Total Environ..

[17] Sha Z (2020). Effect of combining urea fertilizer with P and K fertilizers on the efficacy of urease inhibitors under different storage conditions. J. Soils Sediments.

[18] Barr, D., Garnier, E. & Adom-Owusu, K. Composition containing *N*-(*n*-Butyl) thiophosphoric triamide adducts and reaction products 1–16 (2017).

[19] Loveland Products. Nitrain express 2.0 (2020).

[20] Soares JR, Cantarella H, de Menegale MLC (2012). Ammonia volatilization losses from surface-applied urea with urease and nitrification inhibitors. Soil Biol. Biochem..

[21] Cantarella, H., Soares, J. R., Sousa, R. M., Otto, R. & Sequeira, C. Stability of urease inhibitor added to urea 12–15 (2016).

[22] Santos CF (2021). Dual functional coatings for urea to reduce ammonia volatilization and improve nutrients use efficiency in a Brazilian corn crop system. J. Soil Sci. Plant Nutr..

[23] Engel RE, Towey BD, Gravens E (2015). Degradation of the urease inhibitor NBPT as affected by soil pH. Soil Sci. Soc. Am. J..

[24] Liu S (2019). Ammonia volatilization loss and corn nitrogen nutrition and productivity with efficiency enhanced UAN and urea under no-tillage. Sci. Rep..

[25] Rodella AA, Stipp SR (2018). Requisitos de natureza físico—química. Requisitos de qualidade dos fertilizantes minerais.

[26] Bertin, M. A., Dammann, L., Shirley, A. & van Pol, W. Methods and systems for coating granular substrates 1–15 (2021).

[27] Watson CJ, Akhonzada NA, Hamilton JTG, Matthews DI (2008). Rate and mode of application of the urease inhibitor *N*-(*n*-butyl) thiophosphoric triamide on ammonia volatilization from surface-applied urea. Soil Use Manag..

[28] Lang, T., Zoltan, B., Muelheims, P., Flajs, A. & Denecke, H. Processo para isolamento de um derivado ácido 1–95 (2018).

[29] Li Q (2015). Effect of a new urease inhibitor on ammonia volatilization and nitrogen utilization in wheat in north and northwest China. Field Crops Res..

[30] Santos C (2023). Corn cropping system and nitrogen fertilizers technologies affect ammonia volatilization in Brazilian tropical soils. Soil Syst..

[31] Byrne MP (2020). Urease and nitrification inhibitors—As mitigation tools for greenhouse gas emissions in sustainable dairy systems: A review. Sustainability (Switzerland).

[32] Abalos D, Jeffery S, Sanz-Cobena A, Guardia G, Vallejo A (2014). Meta-analysis of the effect of urease and nitrification inhibitors on crop productivity and nitrogen use efficiency. Agric. Ecosyst. Environ..

[33] Cantarella H, Otto R, Soares JR, de Silva AGB (2018). Agronomic efficiency of NBPT as a urease inhibitor: A review. J. Adv. Res..

[34] Cancellier EL (2016). Ammonia volatilization from enhanced-efficiency urea on no-till maize in Brazilian cerrado with improved soil fertility. Ciência e Agrotecnologia.

[35] Mira AB (2017). Optimizing urease inhibitor usage to reduce ammonia emission following urea application over crop residues. Agric. Ecosyst. Environ..

[36] Li Q (2017). A new urease-inhibiting formulation decreases ammonia volatilization and improves maize nitrogen utilization in North China Plain. Sci. Rep..

[37] Bender RR, Haegele JW, Ruffo ML, Below FE (2013). Nutrient uptake, partitioning, and remobilization in modern, transgenic insect-protected maize hybrids. Agron. J..

[38] European Committee for Standardization. Fertilizers-determination of *N*-(*n*-Butyl)thiophosphoric acid triamide (NBPT) and *N*-(*n*-Propyl)thiophosphoric acid triamide (NPPT)—Method using high-performance liquid chromatography (HPLC) (2015).

[39] ABNT NBR-5774. Determinação-de-acidez-livre-fertilizantes 1–2 (2010).

[40] Tabatabai MA (1977). Effects of trace elements on urease activity in soils. Soil Biol. Biochem..

[41] Kjeldahl J (1883). Neue Methode zur Bestimmung des Stickstoffs in organischen Körpern. Zeitschrift für analytische Chemie.

[42] Embrapa. *Manual de Métodos de Análise de Solo* (2017).

[43] Martins MR (2015). Nitrous oxide and ammonia emissions from N fertilization of maize crop under no-till in a Cerrado soil. Soil Tillage Res..

[44] Pozzi Jantalia C (2012). Nitrogen source effects on ammonia volatilization as measured with semi-static chambers. Agron. J..

[45] R Foundation Statistical Development Core Team. R: A language and environment for statistical computing (2018).

