# Author Correction: Urease inhibitors technologies as strategy to mitigate agricultural ammonia emissions and enhance the use efficiency of urea-based fertilizers

**DOI:** 10.1038/s41598-024-56270-4

**Published:** 2024-03-07

**Authors:** Adrianne Braga da Fonseca, César Santos, Ana Paula Pereira Nunes, Damiany Pádua Oliveira, Maria Elisa Araújo de Melo, Thalita Takayama, Bethânia Leite Mansur, Thales de Jesus Fernandes, Gilson do Carmo Alexandrino, Marcos Altomani Neves Dias, Douglas Guelfi

**Affiliations:** 1 Laboratory of Fertilizers Technologies-INNOVA FERT, Department of Soil Science, Federal University of Lavras-UFLA, P.O. Box 3037, Lavras, MG 37203-202 Brazil; 2 Departament of Statistic, Federal University of Lavras, Lavras, MG Brazil; 3 NUTRIEN, São Paulo, Brazil

Correction to: *Scientific Reports* 10.1038/s41598-023-50061-z, published online 20 December 2023

The original version of this Article contained errors.

In the Materials and Methods section, under the subheading ‘Preparation of fertilizers’,

“**Nitrain technology:** Developed by Loveland products^19^. It is an exclusive blend of NBPT combined to a formulation system of fertilizer biocatalysts. According to the manufacturer, the composition of the Nitrain biocatalyst is similar to ACCOMPLISH LM, including organisms such as *Bacillus pumilus*, *Bacillus megaterium*, and *Bacillus licheniformis *as the main active ingredients^19^. The formulation was applied at a rate of 1.9 kg t^−1^.”

now reads:

“**Nitrain technology:** According to the manufacturer, Nitrain's formulation ensures better nitrogen utilization efficiency, boosting crop growth. This formulation was applied at a rate of 1.9 kg t^−1^.”

In addition, the Funding section was incomplete.

“This work was supported by the Agency for Improvement of Higher-Level Personnel, the National Council for Scientific Development and Technology, and the Minas Gerais.”

now reads:

“This study was conducted with financial support from Nutrien and scholarships granted by the Coordination for the Improvement of Higher Education Personnel (CAPES) and the National Council for Scientific and Technological Development (CNPq).”

The original Article has been corrected.

