# Supplementary material for: Urease inhibitors technologies as strategy to mitigate agricultural ammonia emissions and enhance the use efficiency of urea-based fertilizers
Source: Sci Rep. 2023 Dec 20;13:22739. doi: 10.1038/s41598-023-50061-z (PMC10733344; doi:10.1038/s41598-023-50061-z)
Supplement: Supplementary file 1 — Supplementary Information. [file 41598_2023_50061_MOESM1_ESM.docx]

**Supplementary Materials**

**Table S1**. Climatic data of Lavras, Ingaí, and Luminárias in the first seven days after application of N to the soil.

| Climatic data | Days after N application | | | | | | | |
| --- | --- | --- | --- | --- | --- | --- | --- | --- |
|  | 1 | 2 | 3 | 4 | 5 | 6 | 7 | ∑ 7 days |
|  | --------------------------------------Lavras -------------------------------------- | | | | | | | |
| Precipitation (mm) | 0 | 0 | 0 | 1.2 | 27.6 | 0 | 0 | 28.8 |
| Temperature (max. °C) | 32 | 32.4 | 31 | 31 | 31 | 31 | 32 | - |
| Humidity (%) | 62.5 | 64.5 | 80.5 | 70.25 | 70.25 | 66.5 | 65.2 | - |
|  | --------------------------------------Ingaí ---------------------------------------- | | | | | | | |
| Precipitation (mm) | 0 | 22 | 3 | 0 | 0 | 17 | 0 | 42 |
| Temperature (max. °C) | 28 | 27 | 24 | 25 | 27 | 27 | 26 | - |
| Humidity (%) | 65.5 | 63.5 | 80.5 | 77.5 | 86.5 | 78.5 | 71.5 | - |
|  | ----------------------------------Luminárias ------------------------------------- | | | | | | | |
| Precipitation (mm) | 0 | 6 | 0 | 0 | 7 | 10 | 25 | 48 |
| Temperature (max. °C) | 28 | 27 | 24 | 25 | 27 | 27 | 26 | - |
| Humidity (%) | 31 | 31 | 31 | 31 | 30 | 30 | 30 | - |

**Table S2**. Organic matter content, pH, N mineralization estimate, mineral N stocks, and total soil N in Lavras, Ingaí, and Luminárias.

| Soil depth | Organic matter | pH | N content | | | Soil density | N stock | | | | Estimate of N mineralization and availability | | |
| --- | --- | --- | --- | --- | --- | --- | --- | --- | --- | --- | --- | --- | --- |
|  |  |  | Total | NH_4_^+^ | NO_3_^-^ |  | Total | NH_4_^+^ | NO_3_^-^ | Mineral | Mineralized-N^a^ | Mineral-N^b^ | Available-N^c^ |
| cm | % | CaCl_2_ | g kg^-1^ | ---mg kg^-1^ --- | | g cm^-3^ | ----------------kg ha^-1^ ---------------- | | | | kg ha^-1^ year^-1^ | ------- kg ha^-1^ ------ | |
| ------------------------------------------------------------------------------------ Lavras --------------------------------------------------------------------------------------- | | | | | | | | | | | | | |
| 0-5 | 4.2 | 5.8 | 3.0 | 22.0 | 106.1 | 1.1 | 1798.4 | 13.0 | 62.8 | 75.8 | 42.8 | 75.8 | 118.6 |
| 5-10 | 3.8 | 5.3 | 2.2 | 73 | 94.3 | 1.1 | 1356.4 | 43.6 | 56.3 | 99.9 | 31.2 | 99.9 | 131.1 |
| 10-20 | 2.9 | 5.2 | 1.6 | 41.1 | 78.7 | 1.2 | 1950.5 | 49.3 | 94.5 | 143.5 | 45.0 | 143.5 | 188.5 |
| 20-40 | 2.3 | 5.4 | 1.7 | 26.9 | 77.2 | 1.2 | 4282.0 | 66.1 | 189.7 | 255.8 | 100.6 | 255.8 | 356.4 |
| 0-20 | 3.4 | 5.4 | 2.1 | 44.3 | 89.4 | - | 1763.9 | 63.5 | 77 | 187.4 | 41.0 | 115.6 | 156.5 |
| --------------------------------------------------------------------------------------- Ingaí -------------------------------------------------------------------------------------- | | | | | | | | | | | | | |
| 0-5 | 3.9 | 5.6 | 2.6 | 20.1 | 97.1 | 0.9 | 1237.6 | 9.8 | 46.1 | 55.6 | 29.5 | 55.6 | 85.1 |
| 5-10 | 4.2 | 5.5 | 0.2 | 32.8 | 42.3 | 1.1 | 159.8 | 19.1 | 24.6 | 43.7 | 2.8 | 43.7 | 46.5 |
| 10-20 | 3.0 | 5.7 | 0.2 | 29.3 | 86.5 | 1.1 | 323.0 | 32.2 | 95.2 | 127.4 | 4.8 | 127.4 | 132.2 |
| 20-40 | 2.1 | 5.5 | 1.2 | 29. | 58.7 | 1.0 | 2639.3 | 59.2 | 175.0 | 234.2 | 60.0 | 234.2 | 294.2 |
| 0-20 | 3.5 | 5.6 | 0.8 | 27.8 | 78.1 | - | 540.8 | 23.2 | 65.2 | 88.5 | 10.4 | 88.5 | 99.0 |
| ----------------------------------------------------------------------------------- Luminárias ----------------------------------------------------------------------------------- | | | | | | | | | | | | | |
| 0-5 | 3.6 | 6.4 | 0.2 | 25.1 | 72.2 | 1.0 | 149.2 | 12.7 | 36.7 | 49.4 | 2.4 | 49.4 | 51.8 |
| 5-10 | 3.1 | 5.9 | 0.4 | 25.5 | 73.3 | 1.1 | 242.5 | 15.0 | 43.2 | 58.2 | 4.5 | 58.2 | 62.7 |
| 10-20 | 3.1 | 5.4 | 0.2 | 21.7 | 90.0 | 1.2 | 360.3 | 26.7 | 110.5 | 137.2 | 5.5 | 137.2 | 142.7 |
| 20-40 | 2.1 | 4.7 | 1.4 | 21.1 | 87.4 | 1.2 | 3442.9 | 51.6 | 213.6 | 2652 | 79.3 | 265.2 | 344.5 |
| 0-20 | 3.2 | 5.9 | 0.2 | 23.5 | 81.3 | - | 278.0 | 20.3 | 75.2 | 95.5 | 4.5 | 95.5 | 99.9 |

^a:^ Estimate of annual N mineralization.

^b:^ Values corresponding to the sum of NH_4_^+^ and NO_3_^-^ in N stock.

^c:^ Potentially available N value, as it depends on the N mineralization rate.

**Table S3.** Soil chemical and physical properties at the 0-5, 5-10, 10-20, and 20-40 cm depths in the experimental areas of Lavras, Ingaí, and Luminárias, before the installation of the experiments.

| Soil depth | pH  (CaCl_2_) | K | P-rem | Ca^2+^ | Mg^2+^ | Al^3+^ | (H+Al) | SB | t | T | V | m | Zn^2+^ | Fe^2+^ | Mn^2+^ | Cu^2+^ | B | S-SO_4_ | Sand | Silt | Clay |
| --- | --- | --- | --- | --- | --- | --- | --- | --- | --- | --- | --- | --- | --- | --- | --- | --- | --- | --- | --- | --- | --- |
|  |  | --- mg dm^-3^--- | | ------- cmol_c_ dm^-^³ ------ | | | | -- cmol_c_ dm^-^³ -- | | | ----%---- | | ----------------- mg dm^-3^---------------- | | | | | | ----------%--------- | | |
| ---------------------------------------------------------------------------------------------- Lavras ---------------------------------------------------------------------------------------- | | | | | | | | | | | | | | | | | | | | | |
| 0-5 | 5.8 | 303.6 | 6.5 | 3.5 | 1.6 | 0 | 2.9 | 5.9 | 6.0 | 8.8 | 67.3 | 1.6 | 5 | 53.3 | 11.3 | 1.9 | 0.2 | 12.4 | 22 | 23 | 55 |
| 5-10 | 5.3 | 306.2 | 5.5 | 3.0 | 1.3 | 0.1 | 3.0 | 5.2 | 5.3 | 8.2 | 63.5 | 1.8 | 6.2 | 63.8 | 10.6 | 2.5 | 0.3 | 13.9 | 22 | 24 | 54 |
| 10-20 | 5.2 | 207.4 | 1.0 | 1.8 | 0.8 | 0.1 | 3.2 | 3.2 | 3.2 | 6.4 | 50.5 | 0 | 1.8 | 48.2 | 5.0 | 2.5 | 0.2 | 19.8 | 22 | 21 | 57 |
| 20-40 | 5.4 | 193.3 | 0.7 | 1.4 | 0.6 | 0 | 3.0 | 2.5 | 2.5 | 5.5 | 46.2 | 0 | 1.5 | 48.9 | 4.0 | 2.4 | 0.1 | 18.8 | 21 | 24 | 55 |
| --------------------------------------------------------------------------------------------- Ingaí ---------------------------------------------------------------------------------------------- | | | | | | | | | | | | | | | | | | | | | |
| 0-5 | 5.6 | 255.8 | 15.8 | 3.6 | 1.0 | 0.1 | 5.3 | 5.4 | 5.5 | 10.7 | 50.4 | 1.8 | 2.7 | 54.2 | 12.8 | 0.7 | 0.2 | 27.7 | 31 | 29 | 40 |
| 5-10 | 5.5 | 182.0 | 9.1 | 2.8 | 0.9 | 0.1 | 5.9 | 4.2 | 4.3 | 10.1 | 41.9 | 2.2 | 2.0 | 62.1 | 9.5 | 0.81 | 0.2 | 87.2 | 31 | 22 | 47 |
| 10-20 | 5.7 | 135.7 | 0.5 | 2.4 | 0.9 | 0 | 5.2 | 3.7 | 3.7 | 8.9 | 41.6 | 0 | 1.0 | 58.4 | 5.9 | 0.9 | 0.2 | 133.8 | 29 | 18 | 53 |
| 20-40 | 5.5 | 96.7 | 0 | 1.3 | 0.5 | 0 | 4.3 | 2.1 | 2.1 | 6.4 | 33.1 | 0 | 0.5 | 36.0 | 4.1 | 1.4 | 0.1 | 106.4 | 26 | 15 | 59 |
| ------------------------------------------------------------------------------------------- Luminárias ---------------------------------------------------------------------------------------- | | | | | | | | | | | | | | | | | | | | | |
| 0-5 | 6.4 | 335.4 | 16.7 | 4.6 | 1.3 | 0.1 | 2.1 | 6.9 | 7.0 | 9.0 | 76.7 | 1.4 | 2.5 | 29.2 | 11 | 0.7 | 0.3 | 16.8 | 30 | 30 | 40 |
| 5-10 | 5.9 | 237.0 | 13.4 | 4.0 | 1.1 | 0.1 | 2.9 | 5.7 | 5.8 | 8.6 | 66.4 | 1.7 | 2.2 | 33.7 | 8.3 | 0.9 | 0.2 | 26.5 | 30 | 28 | 42 |
| 10-20 | 5.4 | 153.0 | 4.2 | 2.2 | 0.6 | 0.1 | 4.5 | 3.2 | 3.2 | 7.7 | 41.7 | 0 | 1.4 | 41.7 | 4.1 | 1.0 | 0.1 | 114.3 | 30 | 28 | 42 |
| 20-40 | 4.7 | 119.2 | 0.4 | 1.1 | 0.3 | 0 | 5.0 | 1.8 | 1.8 | 6.8 | 26.5 | 0 | 0.9 | 35.6 | 2.3 | 0.8 | 0 | 193.4 | 30 | 30 | 40 |

pH in CaCl_2_; P, K, Fe, Zn, Mn, and Cu: extractor Mehlich^-1^; Ca^2+^, Mg^2+^, Al^3+^: extractor 1 mol L^-1^ KCl; H + Al: Extractor: SMP; SB: sum of exchangeable bases; CEC (t): Cation Exchange Capacity; CEC (T): Cation Exchange Capacity at pH 7.0; V: Base Saturation Index; m: Aluminum Saturation Index; P-rem: Remaining Phosphorus; B: Extractor–hotwater.

**Table S4.** Urease activity in the three experimental areas during the first seven days after application of different fertilizer technologies in the soil.

| Formulations NBPT^1^ | Urease activity (µg N-NH_4_^+^ g of dry soil h^-1^) | | | | | | | |
| --- | --- | --- | --- | --- | --- | --- | --- | --- |
|  | Day(s) after the application of fertilizer technologies as cover fertilization | | | | | | | |
|  | 0^2^ | 1 | 2 | 3 | 4 | 5 | 6 | 7 |
| ----------------------------------------------------------- Lavras ---------------------------------------------------------- | | | | | | | | |
| SolLC | 1.58 | 0.52 | 0.15 | 1.20 | 0.90 | 2.13 | 0.53 | 1.40 |
| Limus® | 1.89 | 1.17 | 0.75 | 0.58 | 0.54 | 0.84 | 2.05 | 0.58 |
| Nitrain® | 2.40 | 1.48 | 2.19 | 2.25 | 1.46 | 3.38 | 1.74 | 2.71 |
| Anvol® | 1.92 | 1.14 | 1.79 | 2.02 | 2.75 | 3.86 | 1.90 | 0.60 |
| U_GRAN_ | 1.29 | 1.47 | 2.98 | 3.41 | 2.63 | 2.74 | 0.90 | 1.50 |
| Control | 1.31 | 1.29 | 1.48 | 1.92 | 2.14 | 2.55 | 1.85 | 1.75 |
| ----------------------------------------------------------- Ingaí ----------------------------------------------------------- | | | | | | | | |
| SolLC | 1.58 | 0.43 | 1.22 | 1.66 | 1.23 | 1.40 | 1.26 | 0.73 |
| Limus® | 1.53 | 0.62 | 0.83 | 2.26 | 1.55 | 1.32 | 1.15 | 1.04 |
| Nitrain® | 1.87 | 0.65 | 2.59 | 2.22 | 0.89 | 2.16 | 1.45 | 0.74 |
| Anvol® | 1.40 | 1.40 | 1.18 | 1.76 | 1.24 | 2.19 | 0.27 | 1.34 |
| U_GRAN_ | 1.32 | 1.37 | 1.38 | 2.37 | 1.66 | 2.45 | 1.40 | 0.42 |
| Control | 1.59 | 1.39 | 1.71 | 2.1 | 1.65 | 1.80 | 0.85 | 1.58 |
| ------------------------------------------------------- Luminárias -------------------------------------------------------- | | | | | | | | |
| SolLC | 1.79 | 1.75 | 2.17 | 1.42 | 1.11 | 1.77 | 0.59 | 0.95 |
| Limus® | 1.83 | 0.62 | 0.90 | 1.55 | 1.61 | 1.66 | 0.52 | 0.80 |
| Nitrain® | 2.03 | 0.14 | 2.14 | 2.56 | 1.43 | 2.40 | 1.18 | 2.06 |
| Anvol® | 1.50 | 1.21 | 2.42 | 1.63 | 1.15 | 2.00 | 1.56 | 2.03 |
| U_GRAN_ | 1.60 | 1.90 | 2.16 | 1.50 | 1.57 | 2.41 | 1.97 | 1.38 |
| Control | 0.88 | 1.08 | 1.65 | 0.93 | 1.60 | 2.55 | 1.18 | 1.02 |

^1^Urea with and without technologies with N-(n-butyl) thiophosphoric triamide (NBPT) (SolLC, Limus®, Nitrain® e Anvol®). U_GRAN_: granulated urea without NBPT. Control did not receive N fertilization.

^2^Initial condition in the plots before the application the fertilization technologies.

**Table S5.** Daily N-NH_3_ volatilization after 30 days of application of the technologies and U_GRAN_ in the three experimental areas from Brazil.

| Treatment^1^ | Volatilization of N-NH_3_ (%) | | | | | | | | | | | | |
| --- | --- | --- | --- | --- | --- | --- | --- | --- | --- | --- | --- | --- | --- |
|  | Days after application of technologies and U_GRAN_ | | | | | | | | | | | | |
|  | 1 | 2 | 3 | 4 | 5 | 6 | 7 | 9 | 11 | 13 | 15 | 21 | 30 |
| ---------------------------------------------------------------------------------------------- Lavras ------------------------------------------------------------------------------------------- | | | | | | | | | | | | | |
| SolLC | 0.30b | 0.29b | 0.74b | 2.62b | 4.58b | 6.02a | 1.70ab | 4.30a | 1.19ab | 0.44b | 0.08b | 0.13bc | 0.09ab |
| Limus® | 0.24b | 0.39b | 0.31b | 2.78b | 7.24a | 5.26a | 1.63ab | 2.69b | 0.53bc | 0.68a | 0.20ab | 0.24a | 0.08ab |
| Nitrain® | 0.22b | 0.34b | 1.40b | 1.74b | 2.27c | 2.71b | 1.50b | 3.06ab | 0.62bc | 0.30bc | 0.16ab | 0.18ab | 0.10ab |
| SuperN PRO® | 0.23b | 0.40b | 1.32b | 5.12a | 2.50c | 1.78b | 2.46a | 2.79b | 1.62a | 0.22cd | 0.11b | 0.11bc | 0.11a |
| U_GRAN_ | 6.95a | 15.6a | 9.27a | 2.36b | 0.71d | 0.29c | 0.35c | 0.61c | 0.32c | 0.25bcd | 0.31a | 0.11bc | 0.09ab |
| Control | 0.12b | 0.20b | 0.19b | 0.10c | 0.13d | 0.11c | 0.15c | 0.13c | 0.08c | 0.07d | 0.09b | 0.07c | 0.07b |
| ----------------------------------------------------------------------------------------------- Ingaí -------------------------------------------------------------------------------------------- | | | | | | | | | | | | | |
| SolLC | 0.26b | 0.30b | 0.43b | 0.31a | 0.26b | 0.33a | 0.36a | 0.38a | 0.26a | 0.12b | 0.07a | 0.15ab | 0.08a |
| Limus® | 0.36b | 0.35b | 0.41b | 0.53a | 0.65ab | 0.46a | 0.26a | 0.45a | 0.30a | 0.02b | 0.15a | 0.16ab | 0.10a |
| Nitrain® | 0.21b | 0.21b | 0.26b | 0.56a | 0.38b | 0.26a | 1.58a | 0.36a | 0.26a | 0.14ab | 0.10a | 0.12b | 0.10a |
| SuperN PRO® | 0.25b | 0.29b | 0.27b | 0.28a | 0.35b | 0.35a | 0.28a | 0.29a | 0.55a | 0.16ab | 0.12a | 0.20a | 0.09a |
| U_GRAN_ | 17.08a | 13.99a | 3.30a | 0.53a | 1.01a | 0.31a | 0.24a | 0.26a | 0.27a | 0.15ab | 0.13a | 0.14b | 0.09a |
| Control | 1.39 a | 1.71 a | 2.10 a | 1.66 a | 1.80 a | 0.86 ba | 1.58 a | 0.81 b | 2.26 c | 0.42 c | 1.09 cba | 1.26 a | 1.92 a |
| ------------------------------------------------------------------------------------------- Luminárias ---------------------------------------------------------------------------------------- | | | | | | | | | | | | | |
| SolLC | 0.16a | 0.22b | 0.30b | 0.96c | 2.0c | 3.37cd | 4.20a | 5.64b | 1.83a | 0.23a | 0.12a | 0.23a | 0.09a |
| Limus® | 0.18a | 0.34b | 0.25b | 0.85c | 8.9bc | 6.03a | 3.32a | 12.2a | 0.82bc | 0.23a | 0.20a | 0.22a | 0.09a |
| Nitrain® | 0.15a | 0.28b | 0.44b | 1.31c | 4.0dc | 3.98bc | 4.12a | 16.13a | 1.18b | 0.27a | 0.11a | 0.24a | 0.11a |
| SuperN PRO® | 0.13a | 0.25b | 0.38b | 3.03ab | 8.4bc | 5.52ab | 3.72a | 3.38b | 0.76bc | 0.17a | 0.14a | 0.19a | 0.08a |
| U_GRAN_ | 0.21a | 13.6a | 5.81a | 4.06a | 16.4a | 2.10d | 1.89b | 1.03b | 0.47c | 0.12a | 0.15a | 0.12a | 0.09a |
| Control | 1.08 ba | 1.65 a | 0.93 b | 1.60 a | 2.55 a | 1.18 b | 1.02 b | 1.69 a | 0.76 b | 1.60 a | 0.32 b | 1.37 a | 0.77 b |

Means followed by the same letter do not differ according to Tukey’s test (*P* < 0.05).

^1^Urea with and without technologies with N-(n-butyl) thiophosphoric triamide (NBPT) (SolLC, Limus®, Nitrain® e Anvol®). U_GRAN_: granulated urea without NBPT. Control did not receive N fertilization.

**Table S6.** Regression modeled parameters, maximum daily loss by N-NH_3_ volatilization using technologies in relation to U_GRAN_ from the three experimental areas.

| Locations | Formulations  NBPT^1^ | Parameters^2^ | | | | MDL^3^ | Reduction of N-NH_3_ in relation to U_GRAN_ | |
| --- | --- | --- | --- | --- | --- | --- | --- | --- |
|  |  | α (%) | b (dias) | k | R² | (kg N-NH_3_ ha^-1^) | % | (kg N ha^-1^) |
|  | U_GRAN_ | 36.14 | 1.76 | 1.73 | 0.99 | 23.44 | - | - |
| Lavras | SolLC | 22.11 | 5.54 | 0.91 | 0.99 | 7.54 | 38.81 | 21.04 |
|  | Limus® | 21.58 | 5.13 | 1.16 | 0.99 | 9.38 | 40.28 | 21.84 |
|  | Nitrain® | 14.38 | 5.53 | 0.71 | 0.99 | 3.82 | 59.66 | 32.34 |
|  | Anvol® | 18.54 | 5.17 | 0.70 | 0.98 | 4.86 | 48.70 | 26.40 |
|  | U_GRAN_ | 36.67 | 1.06 | 1.68 | 0.98 | 23.10 | - | - |
|  | SolLC | 3.42 | 6.00 | 0.37 | 0.99 | 0.47 | 90.67 | 49.88 |
| Ingaí | Limus® | 3.06 | 5.16 | 0.48 | 0.98 | 0.55 | 91.66 | 50.42 |
|  | Nitrain® | 3.24 | 5.11 | 0.44 | 0.99 | 0.53 | 91.17 | 50.15 |
|  | Anvol ® | 4.19 | 4.90 | 0.49 | 0.98 | 0.77 | 88.57 | 48.12 |
|  | U_GRAN_ | 45.97 | 3.44 | 0.90 | 0.97 | 15.51 | - | - |
|  | SolLC | 19.11 | 6.57 | 0.84 | 0.99 | 6.01 | 58.43 | 40.29 |
| Luminárias | Limus® | 33.63 | 6.23 | 0.88 | 0.99 | 11.09 | 26.84 | 18.51 |
|  | Nitrain® | 32.47 | 6.94 | 0.91 | 0.99 | 11.08 | 29.36 | 20.25 |
|  | Anvol ® | 25.78 | 5.28 | 1.15 | 0.99 | 11.11 | 37.46 | 25.82 |

^1^Urea with and without technologies with N-(n-butyl) thiophosphoric triamide (NBPT) (SolLC, Limus®, Nitrain® e Anvol®). U_GRAN_: granulated urea without NBPT. Control did not receive N fertilization.

^2^*α*: asymptotic value that represents the maximum accumulated loss of N-NH_3_; *b*: abscissa of the inflection point indicating the maximum loss by volatilization. *K*: precocity index that indicates the time needed to reach the maximum accumulated loss (*α*). R²: coeficient of determination.

^3^MDL: maximum daily loss (the highest N-NH_3_ loss occurred in a single day), that is, the inflection point of the curve calculated by the equation $MDL=k*(\alpha/4)$.


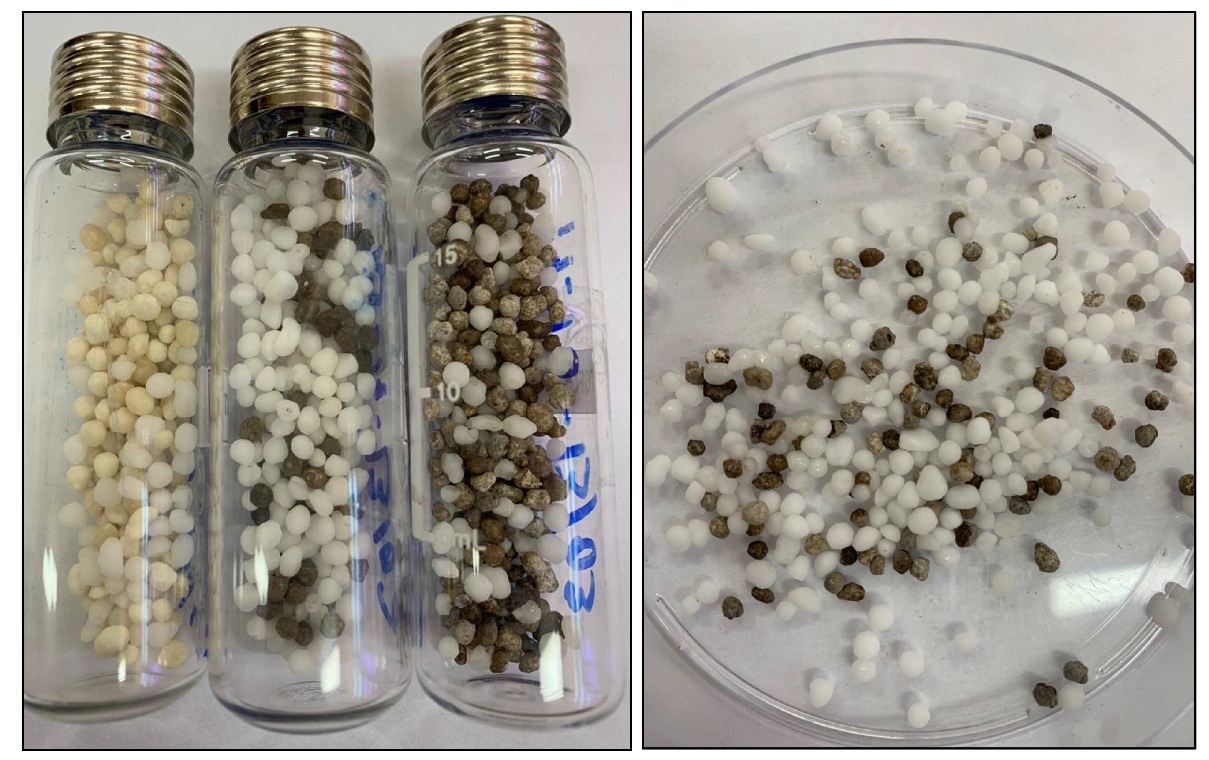


**Figure S1.** Storage test of urea treated with NBPT in combination with conventional phosphate fertilizers (A); Separation of the granules of the fertilizer Urea + NBPT from the phosphate fertilizers, for subsequent quantification of the NBPT after the storage period.


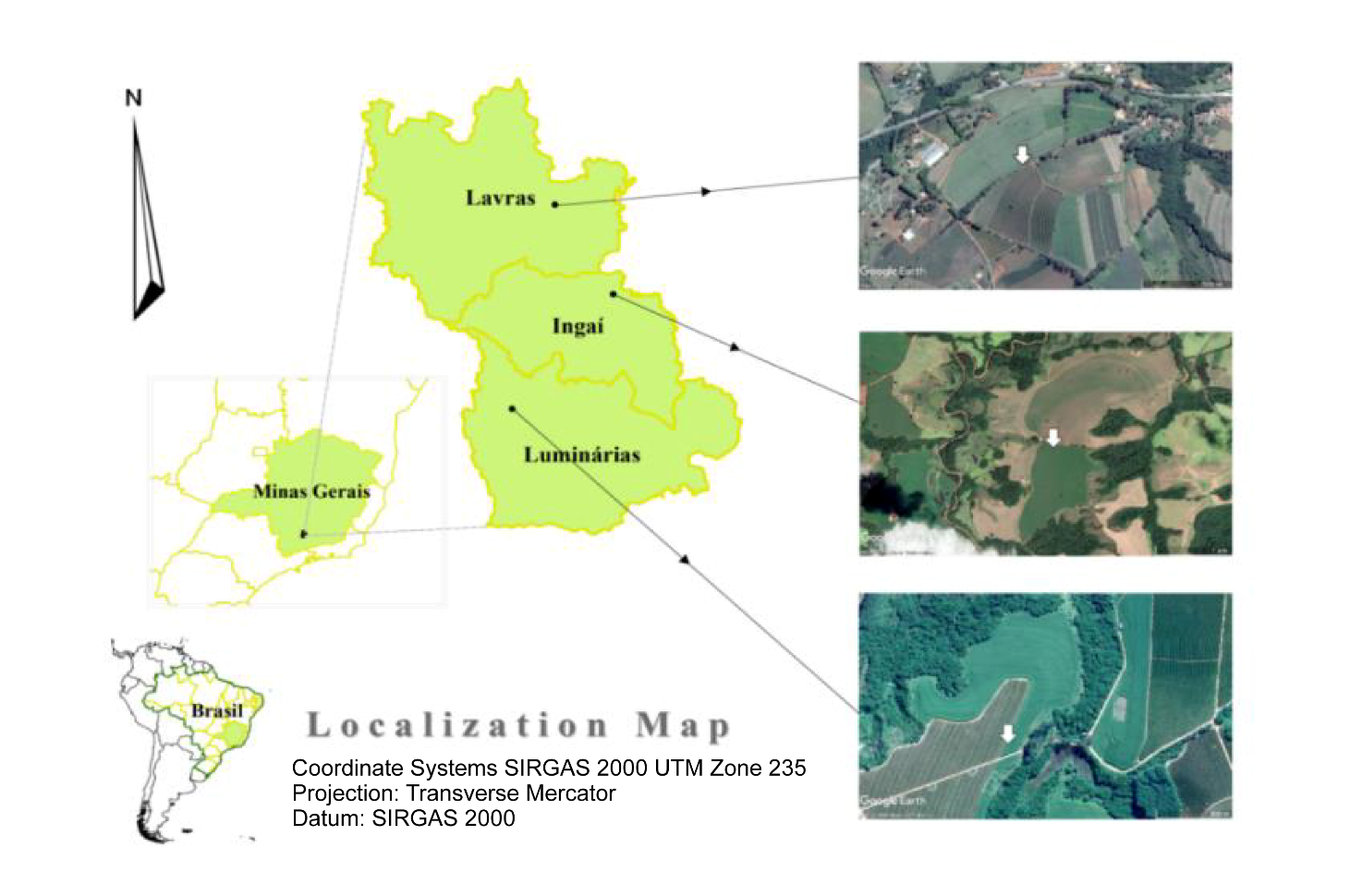


**Figure S2.** Location of experimental areas I, II, and lll, referring to Lavras, Ingaí, and Luminárias municipalities, respectively. Source: **ArcGIS Enterprise 11.0**. An Overview of Map Projections. Available at: **https://webhelp.esri.com/arcgiSDEsktop/9.3/index.cfm?TopicName=An_overvi.**mapa revisado
